# Ammonia emissions from agricultural products at high resolution across Europe

**DOI:** 10.1038/s41597-025-05110-9

**Published:** 2025-08-26

**Authors:** Xinpeng Jin, Paul Behrens, Jan Willem Erisman, José M. Mogollón

**Affiliations:** 1 Institute of Environmental Sciences (CML), Faculty of Science, Leiden University, Leiden, the Netherlands; 2 Oxford Martin School, University of Oxford, Oxford, UK

**Keywords:** Environmental impact, Element cycles

## Abstract

Ammonia (NH_3_) has significant adverse effects on biodiversity, human and ecosystem health. More than 90% of European NH_3_ emissions originate from manure and synthetic fertilizer. Understanding emissions from agricultural products at a high-resolution is essential for environmental policy making. Here, we present an Agricultural, Product-specific, AMMOnia emission dataset (AP-AMMO) for 17 crop groups, 2 grass types, and 6 livestock types across Europe at 5-arc minute resolution. We show that European agriculture emitted 3.5Tg NH_3_-N in 2017, with major livestock products (dairy cattle, other cattle, and swine) and major crop and grassland products (wheat, barley, maize, rapeseed, and permanent grass) contributing 66% and 14%, respectively. When aggregated to national and regional scales, AP-AMMO emissions fall within the range of previous estimates of commonly used (yet more aggregated) models (e.g. EDGAR, GAINS, CLRTAP, IMAGE-GNM, MASAGE). Discrepancies occur due to different excretion and emission factors and the spatial distributions of the production and management of agricultural products. This high-resolution database provides a basis for assessing food system transition impacts on the European nitrogen cycle.

## Background & Summary

Ammonia (NH_3_) is a major form of reactive nitrogen originating mostly from agricultural sources^1,2^ (>90% in Europe) and can lead to many health, biodiversity, and ecosystem harms^3,4^. When NH_3_ enters the atmosphere, it reacts with other pollutants such as NO_X_ or SO_2_, forming particulate matter (PM). The health impacts of particulate matter are substantial, with research suggesting that European NH_3_-driven PM_2.5_ results in 2.6 million years of life lost (YLL) per year, mainly via respiratory and cardiovascular diseases^5^. When NH_3_ is deposited in terrestrial and aquatic ecosystems, it contributes to biodiversity losses and eutrophication^6^. Research shows that nitrogen critical loads for maintaining biodiversity are exceeded across 66% of European agricultural land by NH_3_ deposition only, while the loads for eutrophication are exceeded in over 70% of ecosystem areas by total N deposition (2/3 of which is from NH_3_)^7,8^. Overall, the health costs of NH_3_ in Europe are estimated at 22.9 USD kg^−1^N and biodiversity costs at 4.2 USD kg^−1^ N^5,9^.

Inventories with sufficient spatial, management stage, and product resolution are essential in assessing NH_3_ emissions and possible mitigation options across agricultural regions. Existing datasets do not always include all these criteria simultaneously (Table 1). High-resolution NH_3_ datasets produced by process-based models generally depict the impacts of natural conditions and agricultural measures on N transformations but rarely provide agricultural product-specific estimates^10–12^. High-resolution NH_3_ datasets can also be produced by data-driven models (e.g. mass-balanced models or simplified accounting models)^1,2,13^. These inventories typically have detailed food categories but do not provide consistent estimates of N flows within crop-livestock systems^14^ at the grid level. In addition, some datasets aggregate data to broader sectors or may be inaccessible to the wider community^15^.

**Table 1:** Existing high-resolution ammonia inventories covering Europe and this study for comparison.

Type	Dataset / model	Time	Spatial coverage	Spatial resolution	Mapping method	Accounting level for N flows	Product-specific records	Emission in Europe around 2017 (Tg N)^a^	Data open source
Data driven	CLRTAP grids^40^	1990–2022	51 parties joined in CLRTAP (including Europe)	0.1°lat. × 0.1°lon.	Proxy-based allocation	Tier2	—	2.8 (2017)	Yes
	CAMS-REG-AP^13^	2000–2017	Europe	0.05°lat. × 0.1°lon.	Proxy-based allocation	Tier2	—	3.1 (2017)	Yes
	EDGAR v6.1^1^	1970–2022	Global	0.1°lat. × 0.1°lon.	Proxy-based allocation	Tier1 to 2	—	4.5 (2017)	Yes
	Zhan *et al*.^29^	2000	Global	0.083°lat. × 0.083°lon.	Multi-layer overlap	Tier2	21 crops	0.6 (2000, only from agricultural soils)	Yes
	INTEGRATOR^48^	2010	EU27	1 km × 1 km	Multi-layer overlap	Tier2	4 crop groups and 8 livestock	2.4 (2010, only for EU25)	No
	Yang *et al*.^15^	1961–2018	Global	0.5°lat. × 0.5°lon.	Multi-layer overlap	Tier1	11 crops and 8 livestock	4.0 (2010)	No
	AP-AMMO (This study)	2017	Europe (EU27 + UK + EFTA)	0.083°lat. × 0.083°lon.	Multi-layer overlap	Tier2	17 crops, 2 grasses and 6 livestock	3.5 (2017)	Yes
Process based	FAN v2^10^	2010–2015	Global	2°lat. × 2.5°lon.	Multi-layer overlap	Equals to Tier3	—	3.6 (2010–2015 averaged)	Yes
	MASAGE^41^	2005–2008	Global	2°lat. × 2.5°lon.	Multi-layer overlap	Equals to Tier3	18 crops and 8 livestock	2.7 (2005–2008 averaged)	Yes
	IMAGE-GNM^12,39^	2010	Global	0.5°lat. × 0.5°lon.	Multi-layer overlap	Equals to Tier3	—	2.8 (2010)	Yes

^a^Note: some models/datasets only provide data in earlier years, we choose the emission data in the year nearest to 2017 to fill in this table. The exact time is shown in brackets.

Here, we present the Agricultural Product-specific ammonia emission (AP-AMMO) dataset, a high-resolution (5 × 5 arcmin or 0.083°×0.083°), internally consistent dataset of NH_3_ emissions across European agriculture. It includes 17 crop groups, 2 grass types, and 6 livestock types and is reported for 2017. We developed AP-AMMO by collecting the most recently available agricultural production maps^16–18^ and N flow accounting parameters^19^ (Table S1). We use mass-balanced EEA-Tier 2 approaches^20^ to calculate NH_3_ emissions (reported as NH_3_-N yr^−1^) from synthetic fertilizer and manure. During the NH_3_ accounting, we harmonize data from national inventories^21^, high-resolution maps^18,22^, and regional surveys^23^ for differentiating livestock rearing and manure management systems, linking livestock-specific applied manure N to specific crops, and ensuring data consistency at the grid level. Finally, we compare AP-AMMO against multiple aggregated datasets by stage and agricultural product. We seek to provide a consistent dataset with a high spatial and product resolution to allow integrated assessments of food system transitions, focusing on NH_3_ emissions and their impacts on air quality, nature, and biodiversity protection.

## Methods

### General overview

**Fig. 1:**
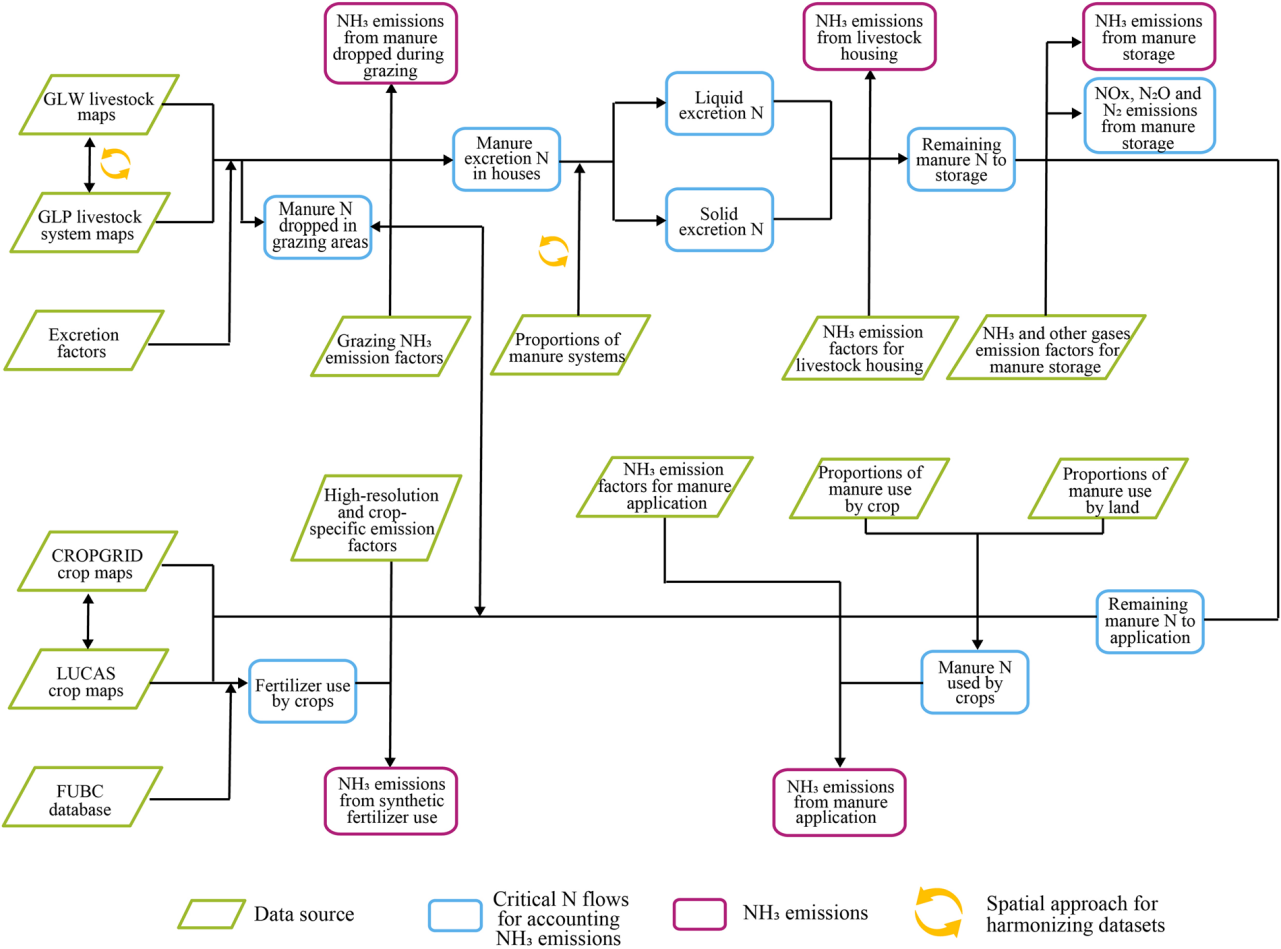
NH_3_ emission accounting model overview.

We present a dataset representing the European agricultural NH_3_ emissions from 25 agricultural products, including 17 crops, 2 grasses and 6 livestock, at a resolution of 5 arc minutes in 2017. For an overview of the modelling framework see Fig. 1 and a list of agricultural products see Table 2. As a basis for the dataset, we take spatial distributions for crop and livestock products mainly from CROPGRIDS^16^ and GLW v4 (Gridded Livestock of the World, version 4)^18^, respectively. We exclude grid cells with agricultural areas (cropland + grassland) of less than 50 hectares (around 1% of the area of a 5-arcminute grid cell in Europe). Further, we collect data to calculate N flows related to NH_3_ emissions. For crop production, we derive fertilizer use by crop (FUBC) data from DRYAD (https://datadryad.org/dataset/doi:10.5061/dryad.2rbnzs7qh)^24^, fertilizer type data from IFASTAT (https://www.ifastat.org/databases), and meteorological data from the CRU TS (https://crudata.uea.ac.uk/cru/data/hrg/)^25^ and Copernicus Climate Change Service (https://surfobs.climate.copernicus.eu/dataaccess/access_eobs.php#datafiles)^26^. To calculate manure N flows, we use national-level data on livestock production and manure systems from National Inventory Reports (NIRs) available through UNFCCC documents (https://unfccc.int/documents) and ClimLPS (Climate and Livestock Production System associated zones) maps^23^. N form and destination data are based on the literature and guidelines, including manure excretion rates from Velthof *et al*.^27^ ammonia nitrogen proportions from the EEA-2019 guideline^20^, and manure application rates from EuropeAgriDB^19^ and Menzi *et al*.^28^ (see Table S1 for detailed information of all data inputs). Finally, we combine the N flows with NH_3_ emission factors from the latest EEA-2019 guideline^20^ (for livestock manure) and a high-resolution emission factor model^29^ for synthetic fertilizer to derive NH_3_ emissions. The main variables and subscripts are summarized in Supplementary Table 1. Our approach inherently preserves the heterogeneity in agricultural productions.

**Table 2:** Agricultural products in this study and their source datasets.

Agricultural products in this study	Agricultural products in source datasets^a^
Wheat	Wheat
Barley	Barley
Grain maize	Maize in LUCAS^17^ and CROPGRID^16^ with proportions of grain maize in Eurostat^33^
Green maize	Maize in LUCAS^17^ and CROPGRID^16^ with proportions of green maize in Eurostat^33^
Rapeseed	Rapeseed
Sunflower	Sunflower
Soybean	Soybean
Oats	Oats
Rice	Rice
Rye	Rye
Potato	Potato
Sugar beet	Sugar beet
Pulses	Bean, chickpea, cowpea, pigeonpea, lentil, bambara, broadbean, lupin, pea, pulsenes, greenbean, greenbroadbean, greenpea, stringbean
Nuts	Almond, cashew, chestnut, hazelnut, kolanut, nutmeg, nutnes, pistachio, walnut
Fruits	Apple, apricot, avocado, berrynes, blueberry, cashewapple, cherry, citrusnes, cranberry, currant, date, fig, fruitnes, gooseberry, grape, grapefruitetc, karite, kiwi, lemonlime, mango, orange, papaya, peachetc, pear, peppermint, persimmon, pineapple, plum, quince, rasberry, sourcherry, stonefruitnes, strawberry, watermelon, tangetc, tropicalnes, coconut, melonetc, melonseed, banana, plantain
Vegetables	Artichoke, asparagus, cabbage, carrot, cauliflower, chicory, cucumberetc, eggplant, garlic, lettuce, mushroom, okra, onion, pepper, pimento, pumpkinetc, spinach, tomato, vegetablenes, vegfor, vetch
Permanent grass	Grassland from LUCAS^17^ and CLMS^35^ with the proportion of permanent grassland to total grassland from Eurostat^33^
Temporary grass	Grassland from LUCAS^17^ and CLMS^35^ with the proportion of temporary grassland to total grassland from Eurostat^33^
Legume fodder	Fodder from LUCAS^17^ with the proportions of alfalfa and other legumes to fodder from Eurostat^33^
Swine	Pigs
Dairy cattle	Cattle from GLW^18^ with the proportions of dairy cattle to total cattle from Eurostat^49^
Non-dairy cattle	Cattle from GLW^18^ with the proportions of non-dairy cattle to total cattle from Eurostat^49^
Poultry	Chicken, ducks
Sheep	Sheep
Goats	Goats

^a^Crop data is from CROPGRID^16^ and livestock data is from GLW^18^ in cells without specific citations.

In the remaining methods section, we report our approach for 1) calculating the *Emissions from manure management* (including livestock housing and manure storage). We then describe 2) the calculations for the *Emissions from synthetic fertilizer use*. Finally, we use the applied manure N from and revised crop maps from last two steps to 3) calculate *Emissions from manure applied and deposited on land*. All NH_3_ emissions and relevant N flows are calculated following the mass-balance based EEA-Tier 2 method^20^, and we report NH_3_ emissions as kg NH_3_-N yr^−1^.

### Emissions from manure management

We first calculate N flows and NH_3_ for livestock housing and manure storage. We trace emissions from manure management for 6 livestock categories: dairy cattle, other cattle, swine, sheep, goats, and poultry. To calculate manure management under different systems we apply livestock quantity and system maps to distinguish grazing and non-grazing animal numbers. Then, for non-grazing animals, we further distinguish liquid and solid manure systems. Finally, we combine livestock system data with livestock numbers with corresponding N flow parameters and emission factors to calculate NH_3_ emissions.

#### Livestock numbers and grazing systems

First, we calculate livestock numbers using the GLW v4 dataset^18^. We further disaggregate dairy and cattle based on the cattle map and their shares at NUTS1 or NUTS2 levels from Eurostat (https://ec.europa.eu/eurostat/web/main/data/database) (Fig. S1). Maps for chicken and ducks were aggregated as poultry. We then rescaled livestock quantities to align with national statistics from FAOSTAT (https://www.fao.org/faostat/en/#data).

Livestock grazing data are originally taken by the GLP dataset^22^ and the GLW dataset^18^. These gridded data are then aligned to grazing data in NIRs^21^ (derived from https://unfccc.int/documents). The GLP provides a layer exclusively distinguishing livestock production systems in each grid cell. When overlaying the GLP layer with GLW maps, we obtain livestock numbers in grazing systems, mixed systems, and other systems. However, the proportion of grazing systems in GLP is land-based, which only accounts for grazing livestock in grassland areas. Meanwhile the NIRs account for grazing animals based on grazing time (that is, grazing animals are not only from grassland systems but also other systems). We follow Vira *et al*.^10^ and allocate a proportion of animals in the mixed systems of the spatial datasets to grazing systems. We define the revised grazing livestock numbers as Equivalent Numbers of Animal being Grazed (*ENAG*). The ENAG in country *g* for livestock *k* based on the combined GLW and GLP spatial dataset (*ENAG_SD*_*k,g*_) is provided by:

1 $${{\rm{ENAG\_SD}}}_{{\rm{k}},{\rm{g}}}={\sum }_{{\rm{i}}=1}^{{\rm{i\; in\; g}}}{{\rm{AMI\_SD}}}_{{\rm{gras\; k}},{\rm{i}}}+{{\rm{AMI\_SD}}}_{{\rm{mix\; k}},{\rm{i}}}\times {{\rm{Prop\_SD}}}_{{\rm{mix\_graz\; i}}}$$

where *AMI_SD*_*gras k,i*_ *and AMI_SD*_*mix k,i*_ are the number of livestock *k* raised in grassland *(gras)* systems and mixed *(mix)* systems at grid cell *i*, respectively. *Prop_SD*_*mix_graz i*_ is the proportion of grazing animals in mixed systems in grid cell *i* and is calculated according to Vira *et al*.^10^. *Prop_SD*_*mix_graz i*_ is temperature based with the assumption that 65% of livestock in mixed systems are grazing when the 10-day running average daily minimum temperature is higher than 10 °C and is given by:

2 $${{\rm{Prop\_SD}}}_{{\rm{mix\_graz\; i}}}={{\rm{Day\_TempH}}10}_{{\rm{i}}}/365\times 0.65$$

where Day_*TempH10*_*i*_ is the number of days whose neighboring ten days’ minimum temperatures are higher than 10°C in grid cell *i*. Daily temperature data are obtained from E-OBS climate data^26^.

Next, we compare *ENAG_SD*_*k,g*_ with equivalent numbers of animals being grazed, *ENAG*, based on NIRs (*ENAG_NIR*_*k,g*_) (Eq. 3). In theory, these estimates should be equal at the aggregated, national level (Eq. 4) and the equations are given by:

3 $${{{\rm{ENAG\_NIR}}}_{{\rm{k}},{\rm{g}}}={\rm{AMI\_NIR}}}_{{\rm{k}},{\rm{g}}}\times {{\rm{Prop\_NIR}}}_{{\rm{graz\; k}},{\rm{g}}}$$

4 $${{\rm{ENAG\_NIR}}}_{{\rm{k}},{\rm{g}}}={{\rm{ENAG\_SD}}}_{{\rm{k}},{\rm{g}}}$$

where *AMI_NIR*_*k,g*_ and *Prop_NIR*_*graz k,g*_ are livestock numbers and corresponding proportions of grazing livestock of livestock *k* in country *g* based on NIRs^21^.

However, discrepancies between *ENAG_SD* and *ENAG_NIR* still exist (Fig. S2). In most cases, *ENAG_SD* is higher than *ENAG_NIR* for cattle, while *ENAG_SD* are lower than *ENAG_NIR* for sheep and goats. We aim to maintain agreement with national totals in order to facilitate the integration of the dataset with national assessments so to address these discrepancies we introduce a rescaling factor (*Fac_scale*_*k,i*_) to balance the two variables:

5 $$\begin{array}{c}{\sum }_{{\rm{i}}=1}^{{\rm{i}}\,{\rm{i}}{\rm{n}}\,{\rm{g}}}{{\rm{A}}{\rm{M}}{\rm{I}}{\rm{\_}}{\rm{S}}{\rm{D}}}_{{\rm{g}}{\rm{r}}{\rm{a}}{\rm{s}}{\rm{k}},{\rm{i}}}+{{\rm{P}}{\rm{r}}{\rm{o}}{\rm{p}}{\rm{\_}}{\rm{S}}{\rm{D}}}_{{\rm{m}}{\rm{i}}{\rm{x}}{\rm{\_}}{\rm{g}}{\rm{r}}{\rm{a}}{\rm{z}}{\rm{i}}}\times {{\rm{A}}{\rm{M}}{\rm{I}}{\rm{\_}}{\rm{S}}{\rm{D}}}_{{\rm{m}}{\rm{i}}{\rm{x}}{\rm{k}},{\rm{i}}}\times {{\rm{F}}{\rm{a}}{\rm{c}}{\rm{\_}}{\rm{s}}{\rm{c}}{\rm{a}}{\rm{l}}{\rm{e}}}_{{\rm{k}},{\rm{i}}}\\ =\,{{\rm{E}}{\rm{N}}{\rm{A}}{\rm{G}}{\rm{\_}}{\rm{S}}{\rm{D}}}_{{\rm{k}},{\rm{g}}}={{\rm{E}}{\rm{N}}{\rm{A}}{\rm{G}}{\rm{\_}}{\rm{N}}{\rm{I}}{\rm{R}}}_{{\rm{k}},{\rm{g}}}\end{array}$$

**Fig. 2:**
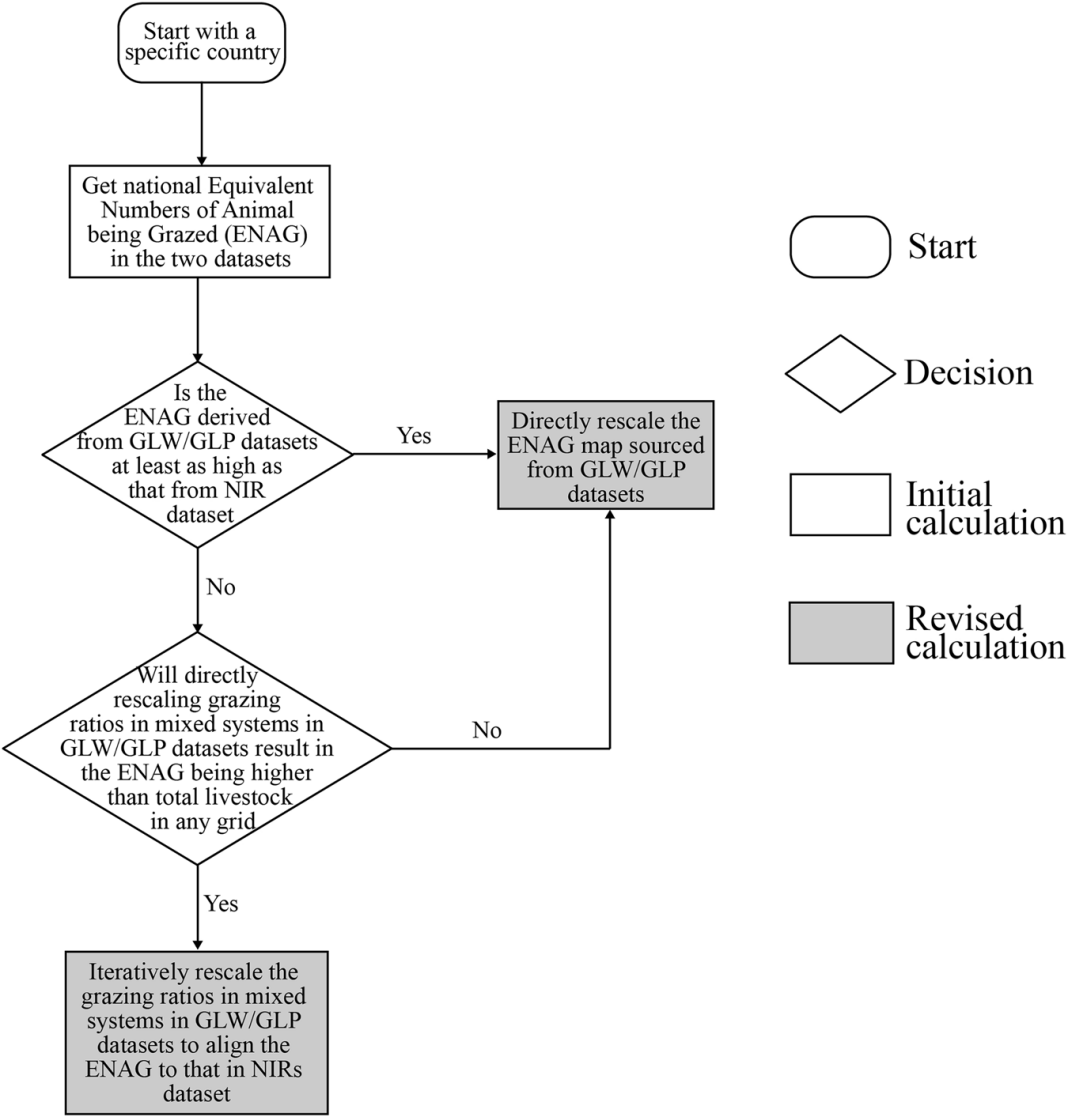
Decision tree for determining the grazing system fractions at the grid level.

We iteratively determine *Fac_scale*_*k,i*_ via a decision tree shown in Fig. 2. When the original *ENAG_SD*_*k,g*_ is higher than *ENAG_NIRFac_scale*_*k,g*_, we apply a lower than 1 for *AMI_SD*_*mix k,i*_ in the whole country *g*, since there is no risk that a revised *ENAG_SD*_*k,g*_ would be higher than all livestock numbers in the original GLW maps in any grid cell. But when the original *ENAG_SD*_*k,g*_ is lower than *ENAG_NIR*_*k,g*_, adopting a factor higher than 1 for all grid cells in a country, may mean that the revised *ENAG_SD*_*k,g*_ exceeds animal numbers in the original GLW maps in some grid cells (Fig. S3).

**Fig. 3:**
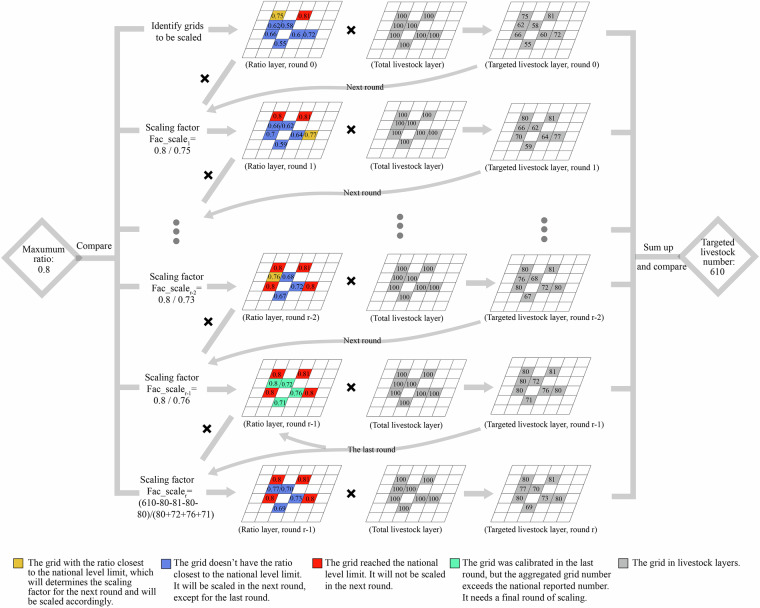
Iterative algorithm for harmonizing grazing animals at the grid level with national data.

We use an iterative solution to limit this factor so that *ENAG_SD* does not exceed total livestock in grid cells while faithfully reproducing the spatial differences in grazing ratios across countries (Fig. 3). We start with assuming that the maximum *Prop_SD*_*mix_graz*_ (*Prop_SD_Max*_*mix_graz*_) in a grid cell of country *g* could be 10%- 20% higher than country averaged *Prop_NIR*_*graz,k,g*_ but must be lower than 100%. We then identify the grid cells having the largest *Prop_SD*_*mix_graz*_ but not exceeding *Prop_SD_Max*_*mix_graz*_ (the yellow cell in the first row of Fig. 3, *Prop_SD_Close*_*mix_graz*_). We obtain an initial *Fac_scale*_*k,i*_ by dividing the *Prop_SD_Max*_*mix_graz*_ by *Prop_SD_Close*_*mix_graz*_. Via *Fac_scale*_*k,g*_, we rescale grid cells where *Prop_SD*_*mix,k,g*_ is lower than *Prop_SD_Max*_*mix_graz*_ (yellow and blue cells in the first row of Fig. 3) and obtain the initial rescaled *ENAG_SD*_*k,g*_ map. If this rescaled *ENAG_SD*_*k,g*_ is still lower than *ENAG_NIR*_*k,g*_, we continue to recalculate *ENAG_SD*_*k,g*_ until it is equal to, or higher than, *ENAG*_*_*_*NIR*_*k,g*_. As a final step, we rescale grid cells values calculated in the penultimate iteration (green grid cells in the fourth row in Fig. 3) with a *Fac_scale*_*k,i*_ lower than or equal to 1 to ensure the final *ENAG_SD*_*k,g*_ *equates to ENAG_NIR*_*k,g*_.

#### Manure management systems

After calculating grazing livestock from grassland and mixed systems, we distinguish manure management for non-grazing animals. We used two datasets to calculate grid-level manure system proportions, NIRs^21^, and ClimLPS^23^. We first classify different manure systems into solids and liquids following Uwizeye *et al*.^30^ (Table S2). Then, we set NIRs data as the benchmark and rescale ClimLPS data to generate regional manure system fraction maps. We use the same iterative rescaling algorithm as in Fig. 3 (the specific decision tree of rescaling livestock number under solid manure systems is shown in Fig. S4).

We can now calculate NH_3_ emissions and N flows through the manure management chain given that we have gridded livestock numbers split into different rearing systems (grazing and non-grazing) and into solid and liquid manure systems.

We trace both N and TAN (Total Ammonia Nitrogen) flows along the manure management chain. NH_3_ and other N gas (N_2_O, N_2_ and NO_x_) emissions are calculated using TAN and total N respectively^20^. We first calculate the manure excretion N and TAN of livestock by rearing system:

6 $${{\rm{N\_Ma}}}_{{\rm{excr\; rsys}},{\rm{k}},{\rm{i}}}=\left\{\begin{array}{cl}{{\rm{ENAG}}{\rm{\_SD}}}_{{\rm{k}},{\rm{i}}}\times {{\rm{EXF}}}_{{\rm{k}},{\rm{g}}} & \,{\rm{if\; rsys}}={\rm{graz}}\\ ({{\rm{ANM}}}_{{\rm{k}},{\rm{i}}}-{{\rm{ENAG}}{\rm{\_SD}}}_{{\rm{k}},{\rm{i}}})\times {{\rm{EXF}}}_{{\rm{k}},{\rm{g}}} & {\rm{if\; rsys}}={\rm{nongraz}}\end{array}\right.$$

7 $${{\rm{T}}{\rm{A}}{\rm{N}}{\rm{\_}}{\rm{M}}{\rm{a}}}_{{\rm{e}}{\rm{x}}{\rm{c}}{\rm{r}}{\rm{r}}{\rm{s}}{\rm{y}}{\rm{s}},{\rm{k}},{\rm{i}}}={{\rm{N}}{\rm{\_}}{\rm{M}}{\rm{a}}}_{{\rm{e}}{\rm{x}}{\rm{c}}{\rm{r}}{\rm{r}}{\rm{s}}{\rm{y}}{\rm{s}},{\rm{k}},{\rm{i}}}\times {{\rm{P}}{\rm{r}}{\rm{o}}{\rm{p}}{\rm{\_}}{\rm{T}}{\rm{A}}{\rm{N}}}_{{\rm{e}}{\rm{x}}{\rm{c}}{\rm{r}}{\rm{k}}}$$

where *N_Ma*_*excr rsys,k,i*_ and *TAN_Ma*_*excr rsys,k,i*_ are total N and TAN in manure excretions of livestock *k* in livestock rearing system *rsys* (e.g. grazing and non-grazing systems) at grid cell *i*; *ANM*_*k,i*_ is the number of livestock *k* at grid cell *i*; *EXF*_*k,g*_ is the excretion factor of livestock *k* in country *g*. *ANM*_*k,i*_ and *ENAG_SD*_*k,i*_ are described fully in the previous section. *EXF*_*k,g*_ is derived from NIRs^21,27^ or, alternatively, from the GAINS model^27,31^ when NIRs are not available. *Prop_TAN*_*excr k*_ is the proportion of TAN to total excreted manure N of livestock *k* derived from EEA guidelines^20^.

Manure TAN (Eq. 8) and N (Eq. 9) excreted in housing are first disaggregate to solid and liquid systems, and then decrease as gaseous losses (including NH_3_ and other nitrous gases) occur in livestock housing and manure storage stages:

8 $${{\rm{T}}{\rm{A}}{\rm{N}}{\rm{\_}}{\rm{M}}{\rm{a}}}_{{\rm{s}},{\rm{m}}{\rm{s}}{\rm{y}}{\rm{s}},{\rm{k}},{\rm{i}}}=\{\begin{array}{c}{{\rm{T}}{\rm{A}}{\rm{N}}{\rm{\_}}{\rm{M}}{\rm{a}}}_{{\rm{e}}{\rm{x}}{\rm{c}}{\rm{r}}{\rm{n}}{\rm{o}}{\rm{n}}{\rm{g}}{\rm{r}}{\rm{a}}{\rm{z}}{\rm{k}},{\rm{i}}}\times {{\rm{P}}{\rm{r}}{\rm{o}}{\rm{p}}{\rm{\_}}{\rm{M}}{\rm{S}}}_{{\rm{m}}{\rm{s}}{\rm{y}}{\rm{s}},{\rm{k}},{\rm{i}}}\,{\rm{i}}{\rm{f}}\,{\rm{s}}=1\\ {{\rm{T}}{\rm{A}}{\rm{N}}{\rm{\_}}{\rm{M}}{\rm{a}}}_{{\rm{s}}-1,{\rm{m}}{\rm{s}}{\rm{y}}{\rm{s}},{\rm{k}},{\rm{i}}}-{{\rm{E}}{\rm{M}}{\rm{I}}{\rm{\_}}{\rm{M}}{\rm{a}}}_{{\rm{s}}-1,{\rm{m}}{\rm{s}}{\rm{y}}{\rm{s}},{\rm{k}},{\rm{i}}}-\\ \,\begin{array}{c}{{\rm{E}}{\rm{M}}{\rm{I}}{\rm{\_}}{\rm{M}}{\rm{a}}{\rm{\_}}{\rm{o}}{\rm{t}}{\rm{h}}}_{{\rm{s}}-1,{\rm{m}}{\rm{s}}{\rm{y}}{\rm{s}},{\rm{k}},{\rm{i}}}\times {{\rm{P}}{\rm{r}}{\rm{o}}{\rm{p}}{\rm{\_}}{\rm{T}}{\rm{A}}{\rm{N}}}_{{\rm{s}}-1,{\rm{k}},{\rm{i}}}\\ \,{\rm{i}}{\rm{f}}\,{\rm{s}}=2\,{\rm{o}}{\rm{r}}\,3\end{array}\,\end{array}$$

9 $${{\rm{N\_Ma}}}_{{\rm{s}},{\rm{msys}},{\rm{k}},{\rm{i}}}=\left\{\begin{array}{c}{{\rm{N\_Ma}}}_{{\rm{excr\; nongraz\; k}},{\rm{i}}}\times {{\rm{Prop\_MS}}}_{{\rm{msys}},{\rm{k}},{\rm{i}}}\,{\rm{if\; s}}=1\\ {{\rm{N}}{\rm{\_Ma}}}_{{\rm{s}}-1,{\rm{msys}},{\rm{k}},{\rm{i}}}-{{\rm{EMI\_Ma}}}_{{\rm{s}}-1,{\rm{msys}},{\rm{k}},{\rm{i}}}-\\ \,{{\rm{EMI\_Ma\_oth}}}_{{\rm{s}}-1,{\rm{msys}},{\rm{k}},{\rm{i}}}\,{\rm{if\; s}}=2\,{\rm{or}}\,3\end{array}\right.$$

10 $${{\rm{Prop\_TAN}}}_{{\rm{s}},{\rm{msys}},{\rm{k}},{\rm{i}}}=\frac{{{\rm{TAN\_Ma}}}_{{\rm{s}},{\rm{msys}},{\rm{k}},{\rm{i}}}}{{{\rm{N\_Ma}}}_{{\rm{s}},{\rm{msys}},{\rm{k}},{\rm{i}}}}$$

where *TAN_Ma*_*s,msys,k,i*_ and *N_Ma*_*s,msys,k,i*_ are manure TAN and N excreted by livestock *k* and managed in manure system *msys* at stage *s* in grid cell *i*. Manure systems *msys* include liquid and solid systems, and stage *s* includes three consecutive stages: housing (*s*=1), storage (*s*=2), and application (*s*=3) for manure excreted by housed animals. *Prop_MS*_*msys,k,i*_ is proportion of manure system *msys* for livestock *k* in grid cell *i*. *EMI_Ma*_*s-1,msys,k,i*_ and *EMI_Ma_oth*_*s-1,msys,k,i*_ are NH_3_ and other types of gaseous emissions (including N_2_, N_2_O, and NO_x_) from the manure managed at the stage *s-1* in manure management system *msys* by livestock *k* in grid cell *i*, respectively. Note that we need to multiply *EMI_Ma_oth*_*s-1,msys,k,i*_ with *Prop_TAN*_*s-1,k,i*_, to ensure we proportionally remove TAN when accounting for the losses from other gasses.

Manure NH_3_ emissions, as well as other gaseous N losses (N_2_, N_2_O and NO_x_) are released from each stage of the manure management chain and are calculated using TAN and N, respectively.

11 $${{\rm{EMI\_Ma}}}_{{\rm{s}},{\rm{msys}},{\rm{k}},{\rm{i}}}={{\rm{TAN\_Ma}}}_{{\rm{s}},{\rm{msys}},{\rm{k}},{\rm{i}}}\times {{\rm{EF\_Ma}}}_{{\rm{s}},{\rm{msys}},{\rm{k}}}\,{\rm{if\; s}}=1\,{\rm{or}}\,2$$

12 $${{\rm{EMI\_Ma\_oth}}}_{{\rm{s}},{\rm{msys}},{\rm{k}},{\rm{i}}}={{\rm{N\_Ma}}}_{{\rm{s}},{\rm{msys}},{\rm{k}},{\rm{i}}}\times {{\rm{EF\_Ma\_oth}}}_{{\rm{s}},{\rm{msys}},{\rm{k}}}\,{\rm{if\; s}}=2$$

where *EF_Ma*_*s,msys,k*_ and *EF_Ma_oth*_*s,msys,k*_ are emissions factors for NH_3_ and other N gasses, respectively. *EF_Ma*_*s,msys,k*_ is derived from EEA-2019 guideline^20^, and *EF_Ma_oth*_*s,msys,k*_ is derived from IPCC guideline^32^ (for N_2_O) and EEA-2019^20^ (for NO_x_ and N_2_).

### Emissions from synthetic fertilizer application

We calculate NH_3_ emissions from synthetic fertilizer for 17 crop groups and 2 grass types, accounting for 95% of harvest area and 90% fertilizer N use across Europe^33,34^. Firstly, we harmonize our crop classification system with those from other sources (Table 2). Then, we collect fertilizer use data from the FUBC dataset (https://datadryad.org/dataset/doi:10.5061/dryad.2rbnzs7qh)^24^, distinguishing fertilizer use for temporary and permanent grasslands following Einarsson *et al*.^19^. Thirdly, we calculate crop-specific high-resolution synthetic fertilizer emission factors according to Zhan *et al*.^29^. With this information in hand, we calculate the total emissions from synthetic fertilizer use.

#### Crop and grass areas

Crop maps are derived from CROPGRIDS dataset^16^, except grasslands and fodder crops are harmonized and reaggregated using the maps from LUCAS (Land Use and Coverage Area frame Survey, https://jeodpp.jrc.ec.europa.eu/services/webview/jeodpp/databrowser/)^17^ and CLMS (Copernicus Land Monitoring Service, https://land.copernicus.eu/en/dataset-catalog)^35^ (Table 2). Maps from LUCAS and CLMS are resampled and aggregated to 5×5 arc minutes.

We disaggregate maize into grain and green maize using the corresponding fractions at NUTS2 (or NUTS1) level from Eurostat^33^ (Fig. S1). For grasses, we first extract the grassland map from LUCAS^17^ (for EU states) and CLMS^35^ (for European Free Trade Association nations). Using the ratio of temporary grass to permanent grass at NUTS1 level from Eurostat^33^, we obtain temporary and permanent grass maps. Legume fodder areas are also obtained from the LUCAS and Eurostat datasets and similarly aggregated to 5×5 arc minutes^17,33^ (Table S3). We also aggregate detailed crop types to pulses, nuts, fruits, and vegetables, aligning with crop categories identified in the FUBC dataset^24^ (Table S4).

#### Fertilizer use by crop and grass

Fertilizer use data in croplands are obtained from the FUBC v9 dataset (https://datadryad.org/dataset/doi:10.5061/dryad.2rbnzs7qh)^24^ with the base year of 2017 (Table S4). While crops defined in our study could find one-to-one matched fertilizer use intensity (fertilizer use per hectare of harvested area) in FUBC v9, this latter dataset does not differentiate between temporary and permanent grassland.

Fertilizer use intensities for permanent and temporary grasses can vary across countries. We distinguish this when accounting for emissions from synthetic fertilizer use. To do this we introduce a ratio of synthetic fertilizer application between permanent grassland and temporary grassland (*Ratio_SF*_*_*_*PermTemp*). According to Einarsson *et al*.^19^, countries in group one (*GF1*) only apply synthetic fertilizer on temporary grassland thus *Ratio_SF*_*_*_*PermTemp* is 0; countries in group two (*GF2*) have defined ratios (*RatioE_SF_PermTemp*); countries in group three (*GF3*) do not have specific ratios (Table S5). We estimate *RatioE_SF_PermTemp* for *GF3* countries by multiplying the ratio of fertilizer use intensity for permanent grassland (*Int_SF*_*_*_*v9*_*permgrass*_) and cropland (*Int_SF*_*_*_*v9*_*cl*_*)* from the FUBC v9 dataset^24^, with those for temporary grassland (*Int_SF_vp*_*tempgrass*_) and cropland (*Int_SF_vp*_*cl*_) from earlier FUBC datasets^24^ (Eq. 13).

13 $${{\rm{Ratio\_SF\_PermTemp}}}_{{\rm{g}}}=\left\{\begin{array}{lc}0 & {\rm{g}}\in {\rm{GF}}1\\ {{\rm{RatioE\_SF\_PermTemp}}}_{{\rm{g}}} & {\rm{g}}\in {\rm{GF}}2\\ \frac{{{\rm{Int\_SF}}}_{{\rm{permgrass\; g}}}}{{{\rm{Int\_SF\_v}}9}_{{\rm{cl\; g}}}}\times \frac{{{\rm{Int\_SF\_vp}}}_{{\rm{cl\; g}}}}{{{\rm{Int\_SF\_vp}}}_{{\rm{tempgrass\; g}}}} & {\rm{g}}\in {\rm{GF}}3\end{array}\right.$$

Following the definitions for fertilizer use for total grassland (*N_SF*_*grass*_), temporary grassland (*N_SF*_*tempgrass*_), and permanent grassland (*N_SF*_*permgrass*_) we solve the synthetic fertilizer allocation to each type of grass (Eqs. 14–16).

14 $${{\rm{N\_SF}}}_{{\rm{grass\; g}}}={{\rm{Int\_SF}}}_{{\rm{perm}}{\rm{grass\; g}}}\times {{\rm{AREA}}}_{{\rm{permgrass\; g}}}+{{\rm{Int\_SF}}}_{{\rm{tempgrass\; g}}}\times {{\rm{AREA}}}_{{\rm{tempgrass\; g}}}$$

We have:

15 $${{\rm{I}}{\rm{n}}{\rm{t}}{\rm{\_}}{\rm{S}}{\rm{F}}}_{{\rm{t}}{\rm{e}}{\rm{m}}{\rm{p}}{\rm{g}}{\rm{r}}{\rm{a}}{\rm{s}}{\rm{s}}{\rm{g}}}=\frac{{{\rm{N}}{\rm{\_}}{\rm{S}}{\rm{F}}}_{{\rm{g}}{\rm{r}}{\rm{a}}{\rm{s}}{\rm{s}}{\rm{g}}}}{{{\rm{R}}{\rm{a}}{\rm{t}}{\rm{i}}{\rm{o}}{\rm{\_}}{\rm{S}}{\rm{F}}{\rm{\_}}{\rm{P}}{\rm{e}}{\rm{r}}{\rm{m}}{\rm{T}}{\rm{e}}{\rm{m}}{\rm{p}}}_{{\rm{g}}}\times {{\rm{A}}{\rm{R}}{\rm{E}}{\rm{A}}}_{{\rm{p}}{\rm{e}}{\rm{r}}{\rm{m}}{\rm{g}}{\rm{r}}{\rm{a}}{\rm{s}}{\rm{s}}{\rm{g}}}+{{\rm{A}}{\rm{R}}{\rm{E}}{\rm{A}}}_{{\rm{t}}{\rm{e}}{\rm{m}}{\rm{p}}{\rm{g}}{\rm{r}}{\rm{a}}{\rm{s}}{\rm{s}}{\rm{g}}}}$$

16 $${{\rm{I}}{\rm{n}}{\rm{t}}{\rm{\_}}{\rm{S}}{\rm{F}}}_{{\rm{p}}{\rm{e}}{\rm{r}}{\rm{m}}{\rm{g}}{\rm{r}}{\rm{a}}{\rm{s}}{\rm{s}}{\rm{g}}}={\rm{R}}{\rm{a}}{\rm{t}}{\rm{i}}{\rm{o}}{\rm{\_}}{\rm{S}}{\rm{F}}{\rm{\_}}{\rm{P}}{\rm{e}}{\rm{r}}{\rm{m}}{\rm{T}}{\rm{e}}{\rm{m}}{\rm{p}}\times {{\rm{I}}{\rm{n}}{\rm{t}}{\rm{\_}}{\rm{S}}{\rm{F}}}_{{\rm{t}}{\rm{e}}{\rm{m}}{\rm{p}}{\rm{g}}{\rm{r}}{\rm{a}}{\rm{s}}{\rm{s}}{\rm{g}}}$$

Where *Int_SF*_*permgrass g*_ and *Int_SF*_*tempgrass g*_ are application intensities of synthetic fertilizer on permanent and temporary grasslands in country *g*, respectively. *AREA*_*pemgrass g*_ and *AREA*_*tempgrass g*_ are areas of permanent grassland and temporary grassland in country *g*, respectively.

#### Crop specific high-resolution emission factors

We obtain high-resolution emission factors of synthetic fertilization for the year 2017 using the regression models of Zhan *et al*.^29^. These models calculate NH_3_ emission factors (*EF_SF*) by modifying baseline emission factors (*EF_SF0*), with modifying functions (***f***) for soil pH (*pH*), air temperature (*Temp*) and wind speed (*Windsd*) during crop growth seasons, and fertilizer application types (*FType*) and methods (*Methd*) (Eq. 17) (detailed function in Eq. 17 is reported in Table S6):

17 $${{\rm{EF\_SF}}}_{{\rm{c}},{\rm{i}}}={{\rm{EF\_SF}}0}_{{\rm{c}},{\rm{i}}}\times f({{\rm{pH}}}_{{\rm{c}},{\rm{i}}})\times f({{\rm{Temp}}}_{{\rm{c}},{\rm{i}}})\times f({{\rm{Windsd}}}_{{\rm{c}},{\rm{i}}})\times f({{\rm{FType}}}_{{\rm{c}},{\rm{i}}})\times {\rm{f}}({{\rm{Methd}}}_{{\rm{c}},{\rm{i}}})$$

All data sources for these parameters consistent with those of Zhan *et al*.^29^ but updated to 2017 (Table S7).

#### Calculating emissions from synthetic fertilization

We obtain crop-specific NH_3_ emissions from synthetic fertilizer via:

18 $${{\rm{E}}{\rm{MI\_SF}}}_{{\rm{c}},{\rm{i}}}={{\rm{A}}{\rm{REA}}}_{{\rm{c}},{\rm{i}}}{\times {\rm{Int}}{\rm{\_SF}}}_{{\rm{c}},{\rm{g}}}\times {{\rm{EF}}{\rm{\_SF}}}_{{\rm{c}},{\rm{i}}}$$

Where *EMI_SF*_*c,i*_ is emissions from synthetic fertilizer use on crop *c* at grid cell *i. AREA*_*c,i*_ is harvested areas of crop *c* at grid cell *i*. *Int_SF*_*c,g*_ is synthetic fertilizer use intensities on crop *c* in country g. *EF_SF*_*c,i*_ is emission factor of synthetic fertilizer use on crop *c* at grid *i*.

### Emissions from manure applied on land and deposited during grazing

Manure N ends up on agricultural land by manure application and/or deposition during grazing. We disaggregate all manure N (see Section *Emissions from manure* *management*) to total cropland and grassland and then to individual grasses and crops according to their areas and manure N input shares by crop and grass. Finally, we calculate emissions from manure deposition and application based on the allocated N flows and corresponding emission factors.

#### Manure N deposited on individual grasslands while grazing

We assume that livestock excretes on grazing areas randomly, thus the livestock excretion goes to individual grasses based on their area shares. The equation is shown as:

19 $${{\rm{N\_Ma}}}_{{\rm{graz\; k}},{\rm{c}},{\rm{i}}}={{\rm{N\_Ma}}}_{{\rm{excr\; graz\; k}},{\rm{i}}}\times \frac{{{\rm{Area}}}_{{\rm{c}},{\rm{i}}}}{{\sum }_{{\rm{c}}}{{\rm{Area}}}_{{\rm{c}},{\rm{i}}}}\,{\rm{c}}\in {\rm{gl}}$$

Where *N_Ma*_*graz k,c,i*_ is the manure N excreted during grazing from livestock *k* to plant *c* at grid cell i. *N_Ma*_*excr graz k,i*_ is manure N excreted during grazing by livestock *k* at grid cell *i* from Eq. 6. *gl* represents grassland, including temporary and permanent grasses.

#### Manure N applied to individual crops and grasses

For manure N applied to cropland, we adopt the approach by Velthof *et al*.^36^ to allocate the manure N to individual crops. Crops are classified into four groups based on assumptions on manure application rates (Table S4). Group One crops are assumed to have largest manure application rates and with manure allocation coefficient of 1, then Group Two to Group Four with the coefficients of 0.5, 0.25 and 0 (0 is for legume crops), indicating the decrease of manure application rates. By combining the manure application coefficients and the areas of individual crops, we can estimate the manure application intensities on each crop group based on Eqs. 20–21:

20 $${{\rm{Int\_Ma\_}}{\rm{A}}{\rm{pp}}}_{{\rm{t}},{\rm{i}}}={{\rm{Coef\_Ma\_allo}}}_{{\rm{t}}}\times {{\rm{Int\_Ma\_}}{\rm{A}}{\rm{pp}}}_{1,{\rm{i}}}$$

21 $${\sum }_{{\rm{t}},{\rm{c}}}{\text{AREA}}_{\text{c},\text{i}}\times {\text{Int\_Ma\_App}}_{\text{t},\text{i}}={{\rm{N}}{\rm{\_}}{\rm{M}}{\rm{a}}}_{{\rm{a}}{\rm{p}}{\rm{p}}{\rm{i}}}\,{\rm{c}}\in {\rm{G}}{\rm{r}}{\rm{o}}{\rm{u}}{\rm{p}}\,{\rm{t}}$$

where *Int_Ma_App*_*t,i*_ is manure N use intensities on crop group *t* at grid cell *i*. *Coef_Ma_allo*_*t*_ is the coefficient of manure N application rates for crop in group *t*. *N_Ma*_*app i*_ is total manure applied on grid cell *i*, which could be acquired from Eq. 9. Note that we wrote all *Int_Ma_App*_*t,i*_ on the basis of manure N use intensities on crop Group One (*Int_Ma_App*_*1,i*_) in Eq.20. This ensures us to solve *Int_Ma_App*_*1,i*_ and *Int_Ma_App*_*t,i*_ under the condition of Eq. 21.

After estimating applied manure N intensities for crop group *t* (*Int_Ma_App*_*t,i*_), we multiply it with individual crop areas belonging to group *t* (*Area*_*c,i*_) to get manure applied on the specific crops. Further, we calculate the manure N applied from specific livestock and manure systems to crops (*N_Ma*_*app msys,k,c*_) by multiplying the manure applied on individual crops with the shares of manure applied by specific livestock and manure systems (Eq. 22).

22 $${{\rm{N}}{\rm{\_}}{\rm{M}}{\rm{a}}}_{{\rm{a}}{\rm{p}}{\rm{p}}{\rm{m}}{\rm{s}}{\rm{y}}{\rm{s}},{\rm{k}},{\rm{c}},{\rm{i}}}={{\rm{I}}{\rm{n}}{\rm{t}}{\rm{\_}}{\rm{M}}{\rm{a}}{\rm{\_}}{\rm{A}}{\rm{p}}{\rm{p}}}_{{\rm{t}},{\rm{i}}}\times {{\rm{A}}{\rm{r}}{\rm{e}}{\rm{a}}}_{{\rm{c}},{\rm{i}}}\times \frac{{{\rm{N}}{\rm{\_}}{\rm{M}}{\rm{a}}}_{{\rm{a}}{\rm{p}}{\rm{p}}{\rm{m}}{\rm{s}}{\rm{y}}{\rm{s}},{\rm{k}},{\rm{i}}}}{{\sum }_{{\rm{k}},{\rm{m}}{\rm{s}}{\rm{y}}{\rm{s}}}{{\rm{N}}{\rm{\_}}{\rm{M}}{\rm{a}}}_{{\rm{a}}{\rm{p}}{\rm{p}}{\rm{m}}{\rm{s}}{\rm{y}}{\rm{s}},{\rm{k}},{\rm{i}}}}$$

#### Emissions from manure inputs from specific livestock to specific crops or grasses

The emissions from livestock manure excreted during grazing are calculated via:

23 $${{\rm{EMI\_Ma}}}_{{\rm{graz\; k}},{\rm{c}},{\rm{i}}}={{\rm{N\_Ma}}}_{{\rm{graz\; k}},{\rm{c}},{\rm{i}}}\times {{\rm{Prop\_TAN}}}_{{\rm{excr\; k}}}\times {{\rm{EF\_Ma}}}_{{\rm{graz\; k}}}\,{\rm{c}}\in {\rm{gl}}$$

where *N_Ma*_*graz k,c,i*_ is manure N deposited during grazing by livestock *k* on plant *c* in grid cell *i*, which is obtained from Eq. 19. *EMI_Ma*_*graz k,c,i*_ is the NH_3_ emissions from *N_Ma*_*graz k,c,i*_. *Prop*_*TAN_Ma*_*excr k*_ is the proportion of TAN to N in manure excretion of livestock *k* in grazing systems. *EF_Ma*_*graz k*_ is emission factor of manure deposition by livestock *k* during grazing. Both *Prop*_*TAN_Ma*_*excr k*_ and *EF_Ma*_*graz k*_ are obtained from the EEA-2019 guideline^20^.

The emissions from livestock manure applied is calculated as:

24 $${{\rm{EMI\_Ma}}}_{{\rm{app\; k}},{\rm{c}},{\rm{i}}}={\sum }_{{msys}}{{\rm{N\_Ma}}}_{{\rm{app\; msys}},{\rm{k}},{\rm{c}},{\rm{i}}}\times {{\rm{Prop\_TAN}}}_{{\rm{app\; msys}},{\rm{k}},{\rm{i}}}\times {{\rm{EF\_Ma}}}_{{\rm{app\; msys}},{\rm{k}}}$$

where *N_Ma*_*app msys,k,c,i*_ is the manure N managed in *msys* systems of livestock *k* and applied on crop *c* in grid cell *i*, which is obtained from Eq. 22. *EMI_Ma*_*app k,c,i*_ is the NH_3_ emission from *N_Ma*_*app msys,k,c,i*_ aggregated by manure systems (*msys*). *Prop_TAN*_*app msys,k,i*_ is the proportion of TAN to N in manure managed in *msys* systems of livestock *k* and appliedd on the cropland in grid *i*, which is from Eq. 10. *EF_Ma*_*app msys,k*_ is the ammonia emission factor of manure managed in *msys* from livestock *k* at the application stage, obtained from the EEA-2019 guideline^20^.

We further aggregate emissions from manure deposition (*EMI_Ma*_*graz k,c,i*_) and application (*EMI_Ma*_*app k,c,i*_) based on the same crop or grassland types to get emissions from manure input from livestock k to plant *c* in grid cell *i (EMI_Ma_Input*_*k,c,i*_):

25 $${{\rm{E}}{\rm{M}}{\rm{I}}{\rm{\_}}{\rm{M}}{\rm{a}}{\rm{\_}}{\rm{I}}{\rm{n}}{\rm{p}}{\rm{u}}{\rm{t}}}_{{\rm{k}},{\rm{c}},{\rm{i}}}=\{\begin{array}{c}{{\rm{E}}{\rm{M}}{\rm{I}}{\rm{\_}}{\rm{M}}{\rm{a}}}_{{\rm{g}}{\rm{r}}{\rm{a}}{\rm{z}}{\rm{k}},{\rm{c}},{\rm{i}}}\,\,\,\,\,\,\,\,{\rm{c}}\in {\rm{c}}{\rm{l}}\\ {{\rm{E}}{\rm{M}}{\rm{I}}{\rm{\_}}{\rm{M}}{\rm{a}}}_{{\rm{g}}{\rm{r}}{\rm{a}}{\rm{z}}{\rm{k}},{\rm{c}},{\rm{i}}}+{{\rm{E}}{\rm{M}}{\rm{I}}{\rm{\_}}{\rm{M}}{\rm{a}}}_{{\rm{a}}{\rm{p}}{\rm{p}}{\rm{k}},{\rm{c}},{\rm{i}}}\,{\rm{c}}\in {\rm{g}}{\rm{l}}\end{array}$$

where *cl* and *gl* represent cropland and grassland, respectively. All other variables were introduced before.

### Emissions from livestock, crop and grassland products

**Fig. 4:**
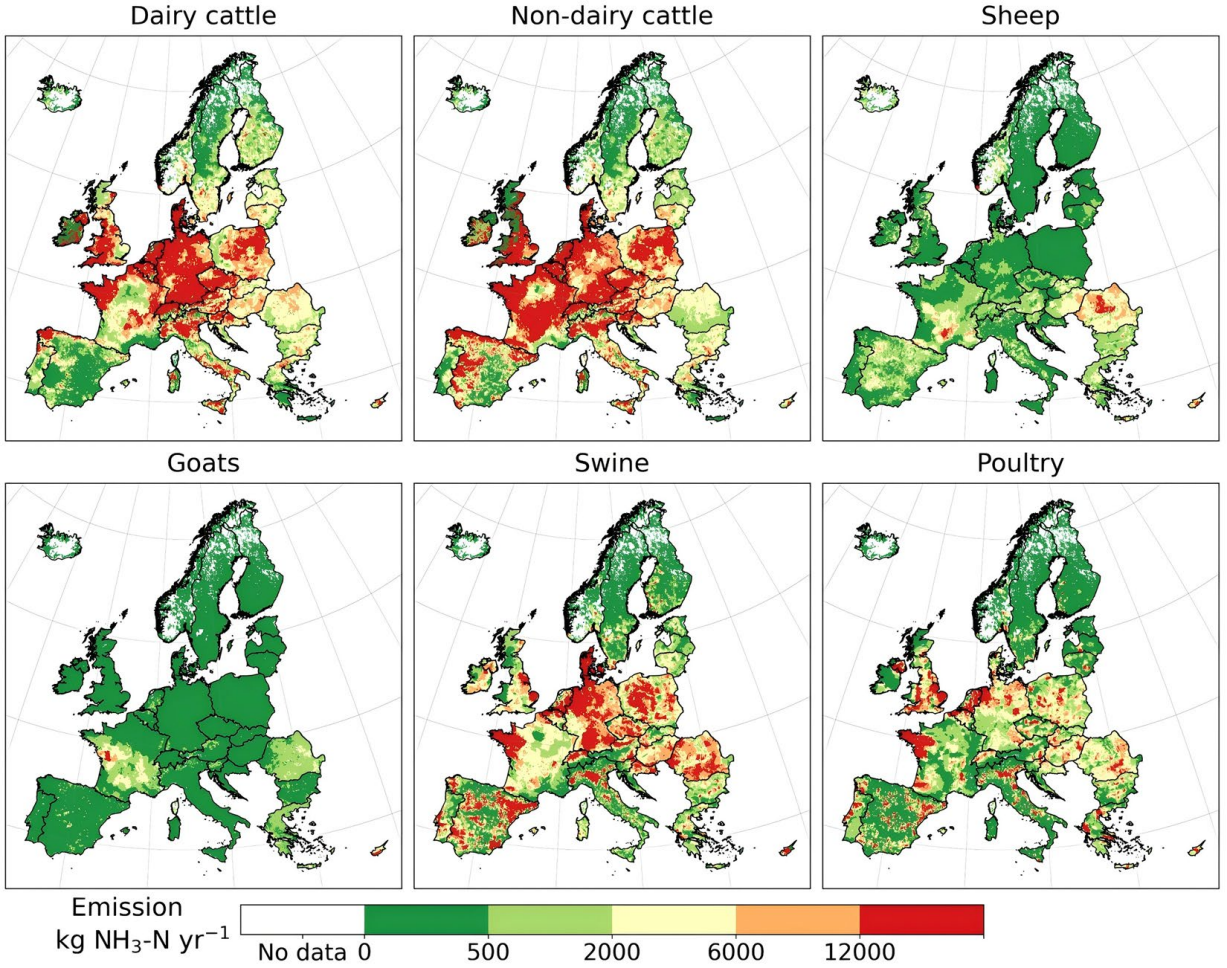
Ammonia emissions along the manure management chain by livestock (including manure deposition during grazing, manure management and manure application).

Ammonia emissions from livestock are aggregated along the manure management chain, including manure management, manure deposited in grazing areas, and manure application. Total NH_3_-N emitted from the manure management chain was 2.81Tg in 2017. Dairy cattle, non-dairy cattle, swine are responsible for 82% of emissions from the manure management chain or 66% of total emissions. Emission distributions are similar for dairy and non-dairy cattle, with hot-spots in the Benelux region, southwestern England, Denmark, northern and southern Germany, central Poland, Austria, Brittany (France), northern Spain, and Italy (Fig. 4). Central France and western Spain are hot-spots only for non-dairy cattle, and Northern Ireland for dairy cattle. Emission hot-spots from swine and poultry have a similar pattern to that of cattle, except with significant hot-spots in southeastern England, inland Spain, and Eastern European countries. Sheep and goats only account for 4% of emissions from the manure management chain. Their emission distributions differ from cattle, as they are more concentrated in the south and east of Europe (Fig. 4).

**Fig. 5:**
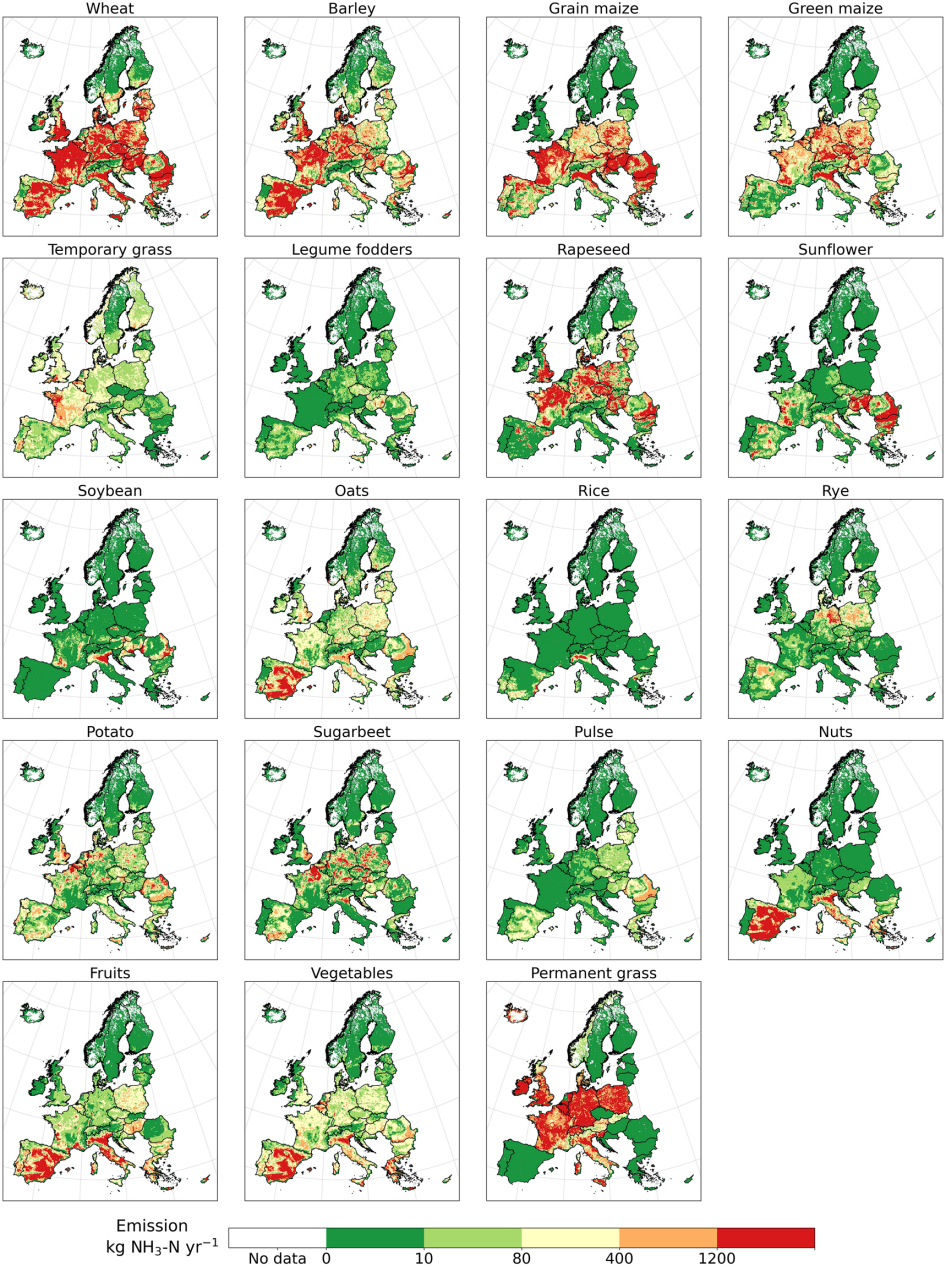
Ammonia emissions from synthetic fertilizer use by crop and grass.

Crops are responsible for 0.71Tg NH_3_-N of synthetic fertilizer use in 2017. The top emitting crop products are wheat, maize, barley, permanent grasses, and rapeseed, accounting for 71% of the crop emissions or 14% of total emissions. Emissions from cereals (wheat, maize, and barley) are prevalent in Europe except for northern Scandinavia and Iceland (Fig. 5). While emissions from permanent grasses are high in western Europe, Poland, and Italy. Additionally, fruits and vegetables contribute to 6% of emission each, mainly located in southern Europe (Fig. 5).

## Data Records

The dataset is available in a figshare repository (10.6084/m9.figshare.27642675)^37^ in.tiff format. We provide NH_3_ emissions for 17 crop groups, 2 grass types, and 6 livestock types by stage. The unit of NH_3_ emissions is reported in kg NH_3_-N yr^−1^, which is in line with the N mass-balance approach we adopted. We cover the EU-27 as well as the UK and European Free Trade Association (EFTA), from latitude 32 N to 72 N, longitude 26.5 W to 36E at the resolution of 5 × 5 arc-minutes.

All files are organized and deposited in folders by stage, namely: synthetic fertilizer use (SF), manure deposition during grazing (MD), manure management (MM), and manure application (MA) (Table 3). These stages correspond to crop or livestock systems and further to specific crop and livestock products. Note that manure input (MI) includes emissions from MD and MA that are allocated to crops, while MD and MA represent those allocated to livestock. File names are recorded as “stage_agricultural product_NH_3_-N.tif” under each folder.

**Table 3:** Data records in AP-AMMO.

Emission stage	Agricultural product group	Individual agricultural product
Synthetic fertilizer use by crops (SF)	Crop and grass	Wheat, barley, maize, green maize, sunflower, soybean, oats, rice, rye, potato, sugar beet, pulses, nuts, fruits, vegetables, temporary grass, permanent grass
Manure input (including manure deposition during grazing and manure application) (MI)		
Manure deposition during grazing (MD)	Livestock	Dairy cattle, non-dairy cattle, sheep, goats, swine, poultry
Manure management (MM)		
Manure application (MA)		

## Technical Validation

We compare our data with existing datasets (from models and inventories) by stage and agricultural product at national level (Table 4). For stage-specific comparisons, we compare with emission inventories that explicitly characterize manure stages (e.g. CLRTAP inventory^2^), process-based models (e.g. FANv2^10^), and MFA models (e.g. EuropeAgriDBv1^19^). There are very few specific datasets on agricultural products, so for these we compare with Zhan *et al*.^29^ for data on emissions from synthetic fertilizer use, and with the GAINS model^38^ for emissions from the manure management chain. We aggregate in various ways (spatial resolution, manure stage) to compare our higher-resolution approach with different studies. Furthermore, we compared emissions from agricultural soils and manure management with those of EDGAR^1^, IMAGE-GNM^39^, and CLRTAP-grid^40^ datasets at the NUTS2 (Nomenclature of Territorial Units for Statistics) level (see Table 1 for information on these datasets).

**Table 4:** Existing datasets used for comparisons.

Comparison type	Comparison items	Datasets
Stage-specific	Emissions per stage	GAINS^38^, CLRTAP^2^, EDGAR^1^, IMAGE-GNM^39^, FANv2^10^, MASAGE^41^, Zhan & Adalibieke^a^^29,44^
	N flows per stage	EuropeAgriDBv1^19^, FAOSTAT^50^, GRAFS^51^, Zhang & Xu^b^^52,53^, Zhan & Adalibieke^29,44^
Product-specific	Emissions by specific livestock product	GAINS^38^
	Emissions by specific crop product	Zhan & Adalibieke^29,44^
Regional	Emissions from agricultural soils and manure management	EDGAR^1^, IMAGE-GNM^39^, CLRTAP-grid^40^

^a^Zhan & Adalibieke dataset is comprised of data from Zhan *et al*.^29^ and Adalibieke *et al*.^44^.They are consecutive studies and from the same research group, so we combined them as one dataset.

^b^Zhang & Xu dataset is comprised of data from Zhang *et al*.^52^ and Xu *et al*.^53^. They are consecutive studies and from the same research group, so we combined them as one dataset.

### Comparisons by ammonia emitting stage

**Fig. 6:**
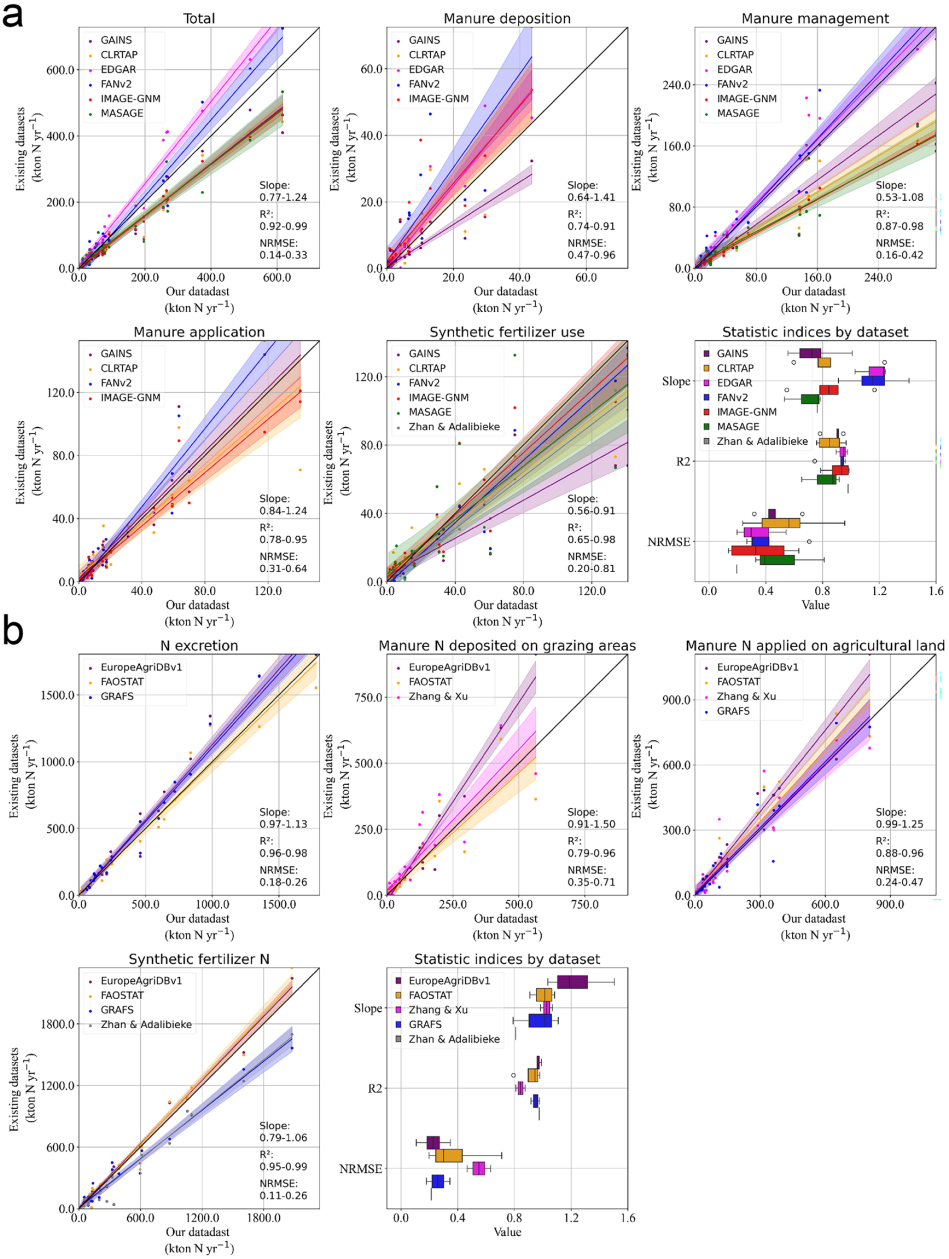
Stage-specific comparisons of (**a**) NH_3_ and relevant (**b**) N flows between our dataset and others at the national level. Data points for each comparison vary from 24 to 32 (total number of countries is 32), depending on data availability at the national level in different datasets. The shadow areas in each panel show 95% confidence intervals of regressions.

Total ammonia (3.5Tg NH_3_-N yr^−1^) in AP-AMMO is within the range of previous datasets (2.7–4.6Tg NH_3_-N yr^−1^). When comparing total emissions with other datasets the R-squared exceeds 0.9, while slopes vary from 0.77 to 1.24. As such, AP-AMMO closely aligns with other datasets with some systematic differences related to emission factors (as statistical indicators show closer alignment when comparing nitrogen flows (Fig. 6b) than emissions (Fig. 6a)).

Regarding single stages, manure management and application emissions exhibit less variability than emissions from manure deposition during grazing and synthetic fertilizer use (Fig. 6a). Manure management emissions show high R-squared (>0.85), low NRMSE (0.16–0.42), and highly variable gradients (0.53 to 1.08) (Fig. 6a), indicating high agreement on emission patterns but large systematic emission differences among datasets. Manure application emissions also have a high agreement with less variable gradients (potentially due to the offset between parameters, that is higher emission factors but less manure quantity at the application stage). Synthetic fertilizer use and manure deposition during grazing have variable gradients, relatively low R^2^ and high NRMSE. This is likely due to different spatial patterns in livestock and crop production. For instance, we aligned gridded grazing animal data with NIRs^21^ at the national level so our emission estimates from manure deposition during grazing align well with GAINS^38^ which has grazing numbers based on NIRs^21^ but show much lower agreement with the FANv2 model^10^ (which uses GLW^18^ grazing numbers and GLP data^22^).

Systematic differences in emissions exist among datasets, primarily due to variations in emission factors and excretion factors. Emissions reported in AP-AMMO show high agreement (R^2^ are high, and slopes are close to one) with EDGAR^1^ and FANv2^10^ which is likely due to similar bottom-up, grid level approaches along with the use of recent emission factors (based on the EEA-2019 guideline^20^). Emissions reported in AP-AMMO are systematically higher than those reported in earlier datasets (MASAGE^41^ and IMAGE-GNM^39^). We find their emission factors and excretion factors are derived from data around 2000^41–43^, whose values are 4–37% and 8–38% lower than ours, respectively. We also see consistently lower emissions (low slopes) in the GAINS model^38^ for the manure management and application stages. This can be attributed to lower excretion factors in GAINS^38^, which are 8–24% lower below those our dataset, along with their lower emission factors incorporated with mitigation measures (Fig. 6a).

### Comparisons by agricultural product

**Fig. 7:**
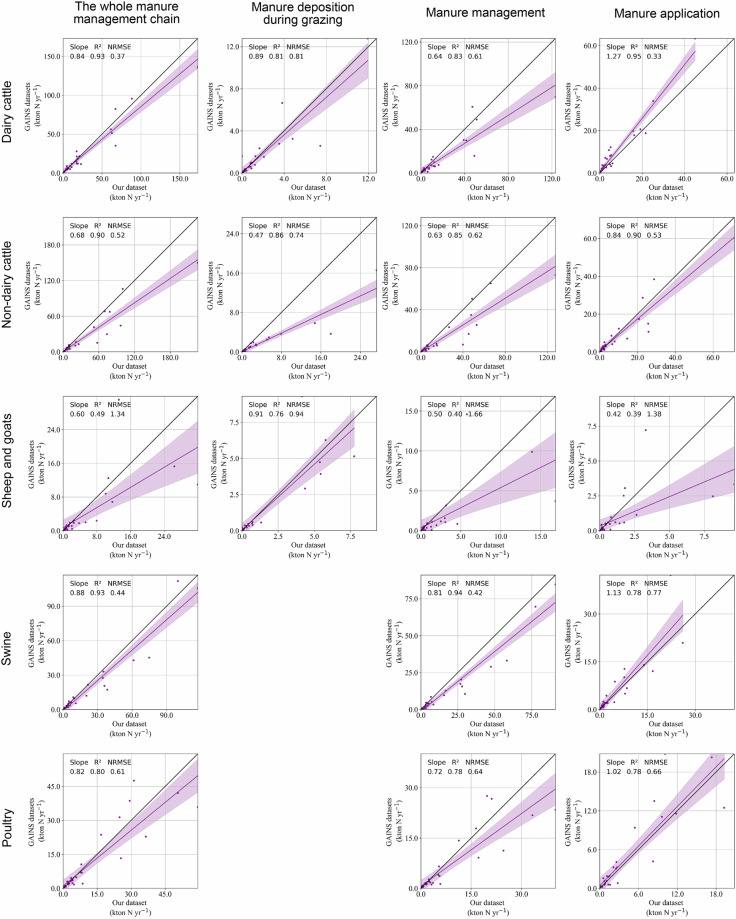
Product- and stage- specific comparisons of NH_3_ emissions between AP-AMMO and the GAINS model^38^ at the national level. Data points for each comparison vary from 30 to 31 (total number of countries is 32) depending on data availability at the national level. The shadow area in each panel shows 95% confidence intervals of the regressions.

The impacts of emission factors, excretion factors, mitigation measures, and animal categorizations on emissions from manure vary depending on specific livestock types. GAINS finds significantly lower emissions from grazing manure deposition by “non-dairy cattle” compared to AP-AMMO (slope = 0.47), again driven by emission factors^38^ (Fig. 7; Fig. S6). Cattle show larger emission variability at the manure management stage (slope < 0.65, R^2^ ≤ 0.85), while swine show more variability at the manure application stage (slope = 1.13, R^2^ = 0.78) (Fig. 7). The former is driven by large difference in excretion factors while the latter is influenced by variations in emission factors due to mitigation effects considered in GAINS^38^ (Fig. S6). Finally, different product categorizations can significantly impact results, for example GAINS^38^ has an aggregated sheep & goats category while we split these animals and use livestock-specific parameters from NIRs^21,27^. This leads to very low R^2^ and slopes (both lower than 0.5) in the comparisons of emissions from manure management and application.

**Fig. 8:**
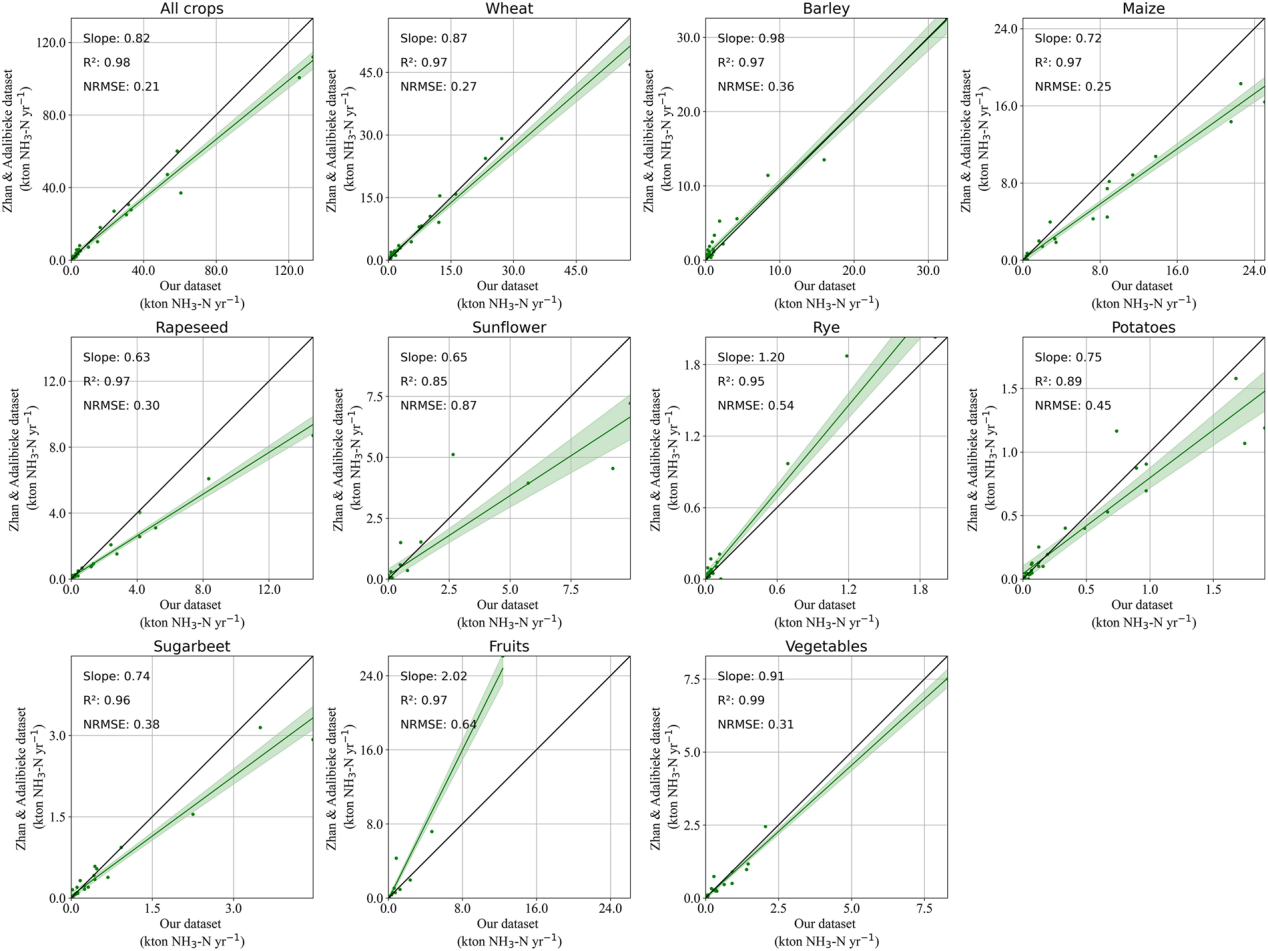
Product-specific comparisons of NH_3_ emissions from synthetic fertilizer use between AP-AMMO and Zhan & Adalibieke^29,44^ at the national level. Data points for each comparison vary from 18 to 29 (total number of countries is 32), depending on data availability at the national level for different crops. The shadow area in each panel shows 95% confidence intervals of the regressions.

Our emissions from synthetic fertilizer on individual crops show high agreements with Zhan & Adalibieke^29,44^, except for fruits (slope is over 2) (Fig. 8). The systematic difference for fruits is largely caused by different synthetic fertilizer intensities (fertilizer intensity in Zhan & Adalibieke is twice ours)^44^ (Fig. S7). Other crops have smaller variabilities between the two datasets (slopes:0.63–0.98, R^2^: 0.85–0.99) (Fig. 8), in which temperature is likely an important factor causing emission difference via influencing emission factors. This could explain why crops growing in warmer months (e.g. maize, sunflower, potatoes, and sugar beet) see larger emission differences (lower slopes and higher NRMSE compared to other crops).

### Comparisons at the NUTS2 level

**Fig. 9:**
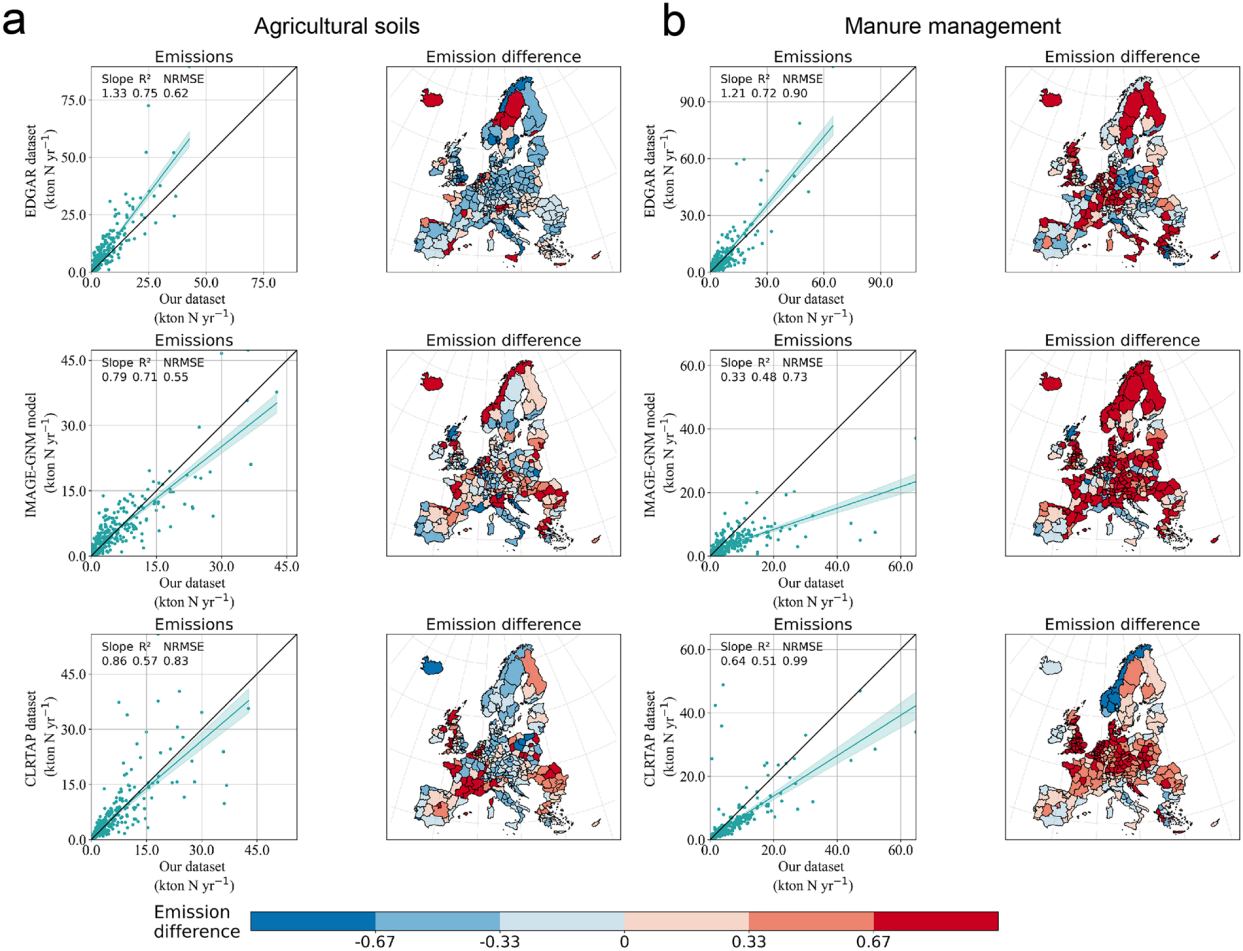
NUTS2 level comparisons for ammonia emissions from (**a**) agricultural soils and (**b**) livestock management with EDGAR^1^ (the first row), IMAGE-GNM^39^ (the second row), and CLRTAP-grid^40^ (the third row). Data points for each comparison vary from 269 to 292 (total number of NUTS2 regions is 295), depending on data availability at the NUTS2 level from different datasets. The shadow areas in comparison panels show 95% confidence intervals of regressions. The emission difference is calculated as the proportion of emission quantity difference between AP-AMMO and other datasets to emissions of AP-AMMO. Positive values indicate that emissions in AP-AMMO are higher than in other datasets, while negative values indicate lower emissions.

Emissions among datasets at the NUTS2 level further misalign, with R^2^ decreasing, NRMSE increasing, and slopes showing greater variabilities compared to national level comparisons (Fig. 9). The relative emission differences, calculated as the ratio of the emission quantity difference between AP-AMMO and other datasets to the emissions of AP-AMMO, also vary significantly at the NUTS2 level.

Here, the spatial distributions of agricultural products and parameters play an important role in comparisons. Agricultural soil emissions in AP-AMMO are significantly lower than in EDGAR^1^ across main crop-producing regions (>33%, shown as sky blue and deep blue regions) (see Fig. 9a). This is likely due to the spatial allocation method of EDGAR^1^, which is based solely on cropland area without considering the varying fertilizer use intensities of different crops. In contrast, AP-AMMO shows higher emissions from soils in high livestock density areas, such as Netherlands and Belgium (likely stemming from our assumption that manure is applied within the grids and areas with high livestock densities may apply more manure than the limit in Nitrate Directive^45^). For manure management emissions, AP-AMMO sees consistently higher values in the Netherlands, southern Germany, south-central France, and southwestern England (>33%, light coral and deep red regions) (Fig. 9b). Other than emission factors, grazing ratios and manure system allocations contribute to differences. For example, we allocate fewer dairy cattle to grazing systems in Germany, the Netherlands and the UK (in line with NIR data^21^), which increases the housed dairy cattle and subsequent emissions. Lastly, Scandinavian emissions vary depending on grassland and livestock distributions. In these regions, AP-AMMO sees higher alignment with IMAGE-GNM^39^ and CLRTAP-grid^40^ for soil emissions and with CLRTAP-grid^40^ from manure management emissions (except Norway, with high emissions). The way grassland layers are explicitly mapped in IMAGE-GNM^39^ and livestock layers are mapped in CLRTAP-grid^40^ may explain these results.

## Usage Notes

We provide a grided NH_3_ emission dataset for Europe adopting EEA-Tier 2^20^ approaches at the agricultural product level (Table 3). We develop spatial approaches for harmonizing existing parameters. Our dataset is agricultural product specific, mass balanced, and internally consistent.

Our dataset has some limitations and strong assumptions that should be considered when reusing it. (1) We disaggregate national reported grazing ratios and manure system ratios to grid level based on existing grided (GLW/GLP)^18,22^ and regional (ClimLPS) data^23^ (Fig. 3). This assumes that the distributions of these parameters largely depend on temperature and the original grid distributions, which needs to be validated by on-the-ground regional data as more farm reporting becomes available^46^. (2) High-resolution emission factors for synthetic fertilizer are estimated using regression models based on global data in Zhan *et al*.^29^. These can be updated as more Europe-specific data become available. (3) Due to a lack of data, we have to sort manure application intensity rations to three groups (high, middle, and low intensity groups) based on previous work^36^ which can also be updated as more data become available. (4) We assume manure is applied within the grid cells where it is produced. For areas with high livestock density and manure production, such as the Netherlands, Flanders, Western Germany and the Po Valley in Italy, this may result in higher manure application than allowed in the Nitrate Directive with a limit of manure application of 170 kgN ha^−1^ and 250 kgN ha^−1^ in derogation regions^45^. These are regions that have long struggled with meeting the directive, however future models could include manure transport and processing to account for situations where excess manure is addressed through other means than local application. (5) Our emission factors for livestock are obtained from EEA- 2019 guidelines^20^ and may not reflect recent local improvements that deviate from those emission factors. Further integrating comprehensive *in-situ* measurement data can improve this aspect in the future^46,47^.

Our dataset is suitable for top-down assessments using large-scale data to provide a comprehensive view of environmental impacts across regions. It can also be used to examine how food system transitions can impact NH_3_ emissions. Future work could explore consumption-based transformations with different livestock-crop production interactions and identify synergies and trade-offs of mitigation measures in nitrogen hot-spots.

## Supplementary information

Supplementary Information

Supplementary Table 1

## Data Availability

Codes in Python used to calculate agricultural product-specific ammonia emissions are available at GitHub:(https://github.com/Xinpeng0930/Code_for_AP_NH3).
