# Supplementary material for: Ammonia emissions from agricultural products at high resolution across Europe
Source: Sci Data. 2025 Aug 26;12:1493. doi: 10.1038/s41597-025-05110-9 (PMC12381134; doi:10.1038/s41597-025-05110-9)
Supplement: Supplementary file 1 — Supplementary Information [file 41597_2025_5110_MOESM1_ESM.docx]

**Table S1** Detailed information for datasets used in this study

| Data categories | Data | Datasets | Geo-spatial information | Product resolution |
| --- | --- | --- | --- | --- |
| Activity data | Crop harvested areas | CROPGRID^1^  (circa 2020) | Global, 0.05°， | Crop specific |
|  |  | LUCAS (Land Use and Coverage Area frame Survey)^2^  (2018) | EU27 + the UK, 100m | Crop specific |
|  |  | CLMS (Copernicus Land Monitoring Service)^3^  (2015) | Europe,100m | Distinguishes cropland and grassland |
|  | Livestock numbers | GLW v4 (Gridded Livestock of the World, version 4)^4^  (2015) | Global, 0.083° | Livestock specific |
|  | Synthetic fertilizer use | FUBC (Fertilizer use by crop)^5^  (1978-2018) | Global main fertilization countries, national level | Crop specific |
|  |  | EuropeAgriDB v1 (1961-2019)^6^ | 26 European countries, national level | Distinguishes cropland and grassland |
| Intermediate parameters | Livestock systems | GLP v5 (Global Livestock Production System, version 5)^7^  (no specific year) | Global,0.083° | For ruminants |
|  |  | NIR (National Inventory Reports)^8^  (2016 or 2017) | Annex I countries, country-level, | Livestock specific: |
|  | Manure systems | NIR ^8^  (2016 or 2017) | As stated above | As stated above |
|  |  | ClimLPS report (Climate and Livestock Production Systems)^9^ (2008) | EU28 (former) plus EFTA excluding Iceland, NUT2-level, | Livestock specific |
|  | Manure excretion factors | NIR^8^  (2016 or 2017) | As stated above | As stated above |
|  |  | GAINS model (Greenhouse Gas and Air Pollution Interactions and Synergies)^10,11^  (2010) | 38 countries in Europe, national level | Livestock specific |
|  | Manure TAN proportions | EEA-2019 guideline^12^  (After 2008) | Europe, European level | Livestock specific |
|  | Manure application rates | INTEGRATOR model (Integrated Nitrogen Tool across Europe for Greenhouse gases and Ammonia Targeted to Operational Responses)^13,14^ (1996) | 30 countries in Europe, national-level | Livestock specific |
|  | Manure allocating ratios | MITERRA-EUROPE model^15^ (No specific year) | EU27, European level | Crop-group specific |
| Emission factors | Manure deposition | EEA-2019 guideline^12^  (After 2008) | As stated above | Livestock specific |
|  | Livestock housing | EEA-2019 guideline^12^  (After 2008) | As stated above | Livestock specific |
|  | Manure storage | EEA-2019 guideline^12^  (After 2008) | As stated above | Livestock specific |
|  | Manure application | EEA-2019 guideline^12^  Sommer et al.^16^  (After 2008) | Europe, European level distinguished by temperature zones | Livestock specific |
|  | Synthetic fertilizer use | High-resolution cropland emission factors model^17^ | Global, 0.083°  (updated with data in 2017) | Crop specific |


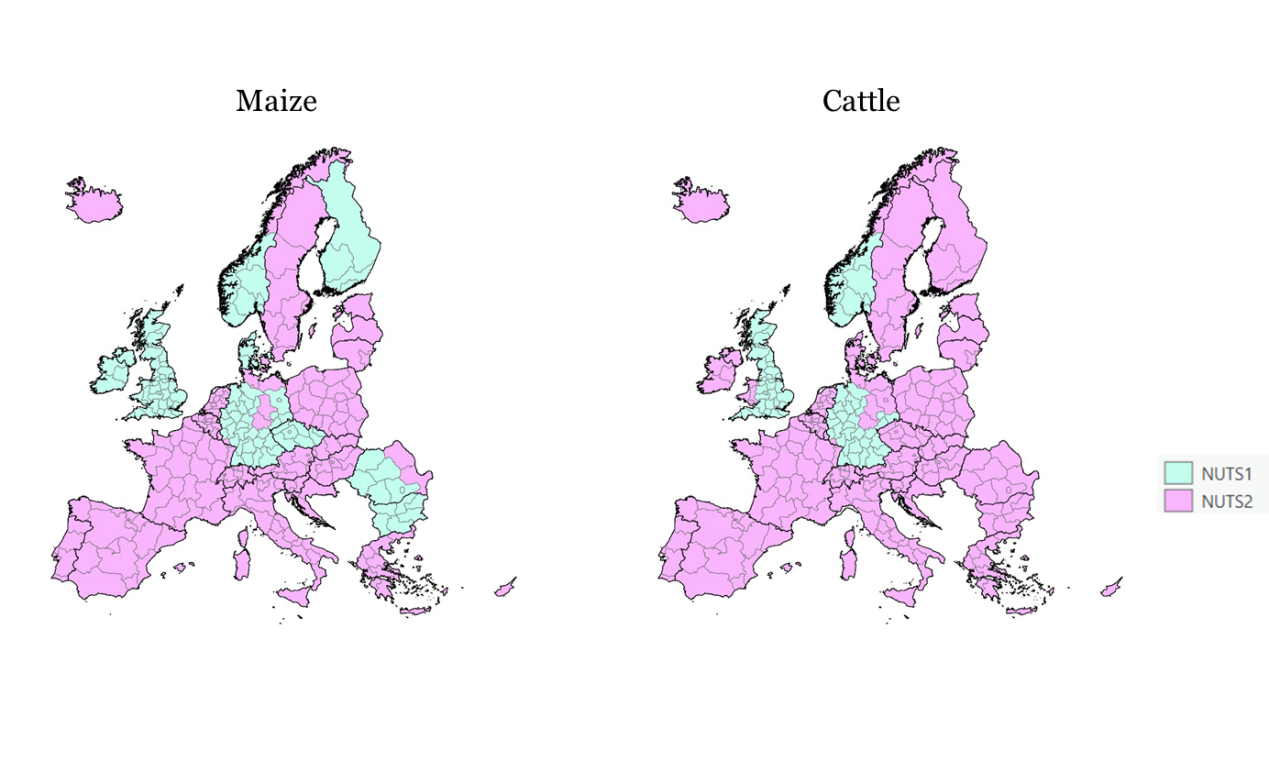


**Fig S1** Disaggregation levels for cattle and maize


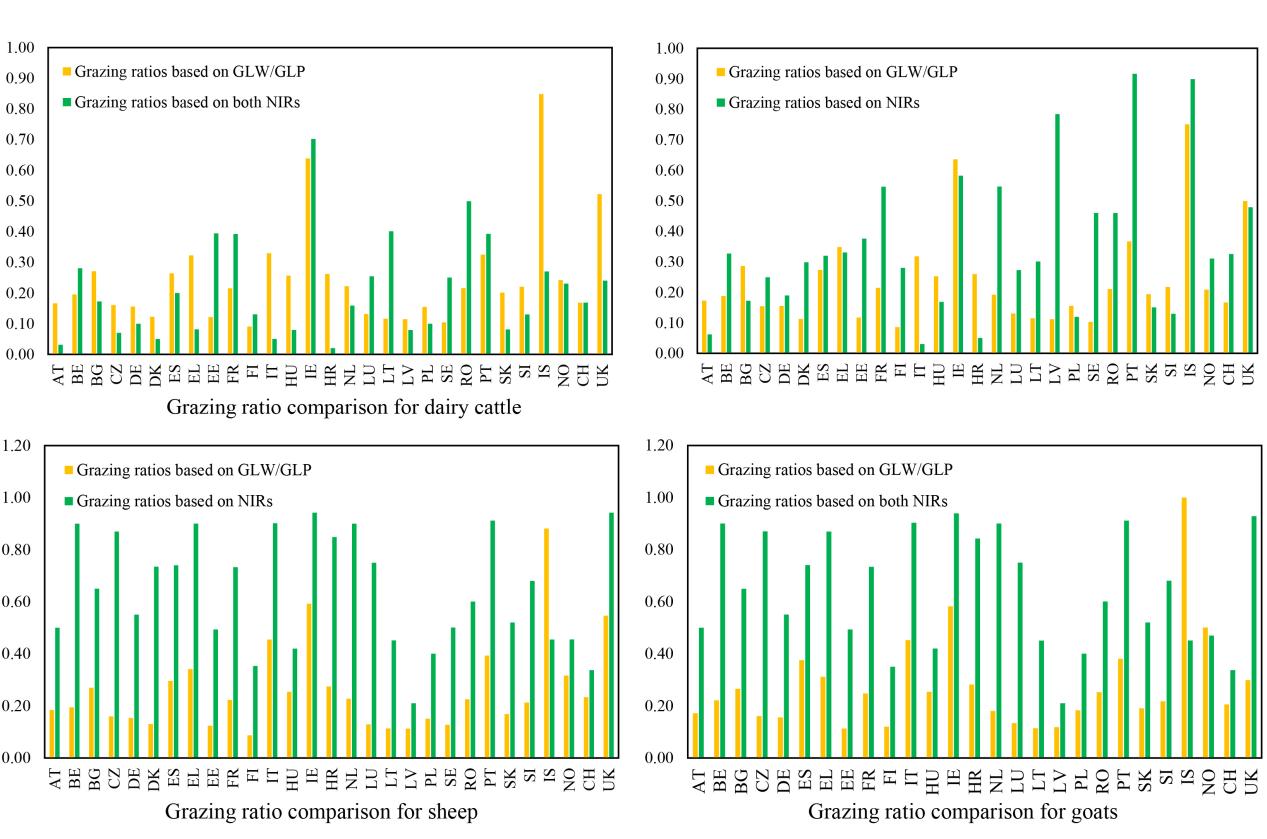


**Fig. S2** Comparisons of grazing ratios between GLW/GLP^4,7^ and NIR^8^ datasets


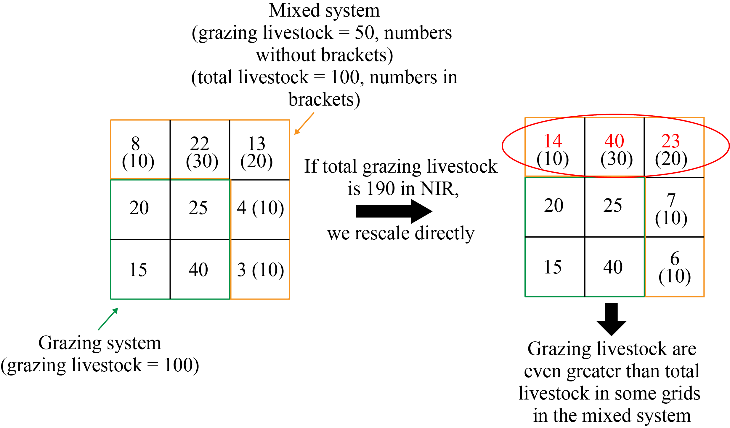


**Fig. S3** An example of rescaling dilemmas when aligning gridded grazing animals to those in NIR datasets

**Table S2** Livestock manure system categorization (adapted from Uwizeye et al.^18^)

| Manure management systems of non-grazing animals in NIR reports | Manure system categories in our study |
| --- | --- |
| Aerobic Treatment | Liquid |
| Anaerobic digestion | Liquid |
| Anaerobic lagoon | Liquid |
| Composting | Solid |
| Deep litter | Solid |
| Digester | Liquid |
| Dry lot | Solid |
| Liquid with crust | Solid |
| Liquid/Slurry | Liquid |
| Manure with litter (poultry) | Solid |
| Manure without litter (poultry) | Solid |
| Solid | Solid |
| Solid storage | Solid |
| Confinement | Liquid for dairy and other cattle in feedlot systems; Solid for dairy and cattle excluding those in feedlot systems, sheep, goats, swine and poultry |
| Daily spread | Liquid for dairy cattle in mixed systems and swine; solid for dairy cattle excluding those in mixed systems, other cattle, sheep, goats and poultry |
| Pit | Liquid for dairy cattle, other cattle, sheep, goats, swine, poultry excluding those intensive systems; solid for poultry in intensive systems |


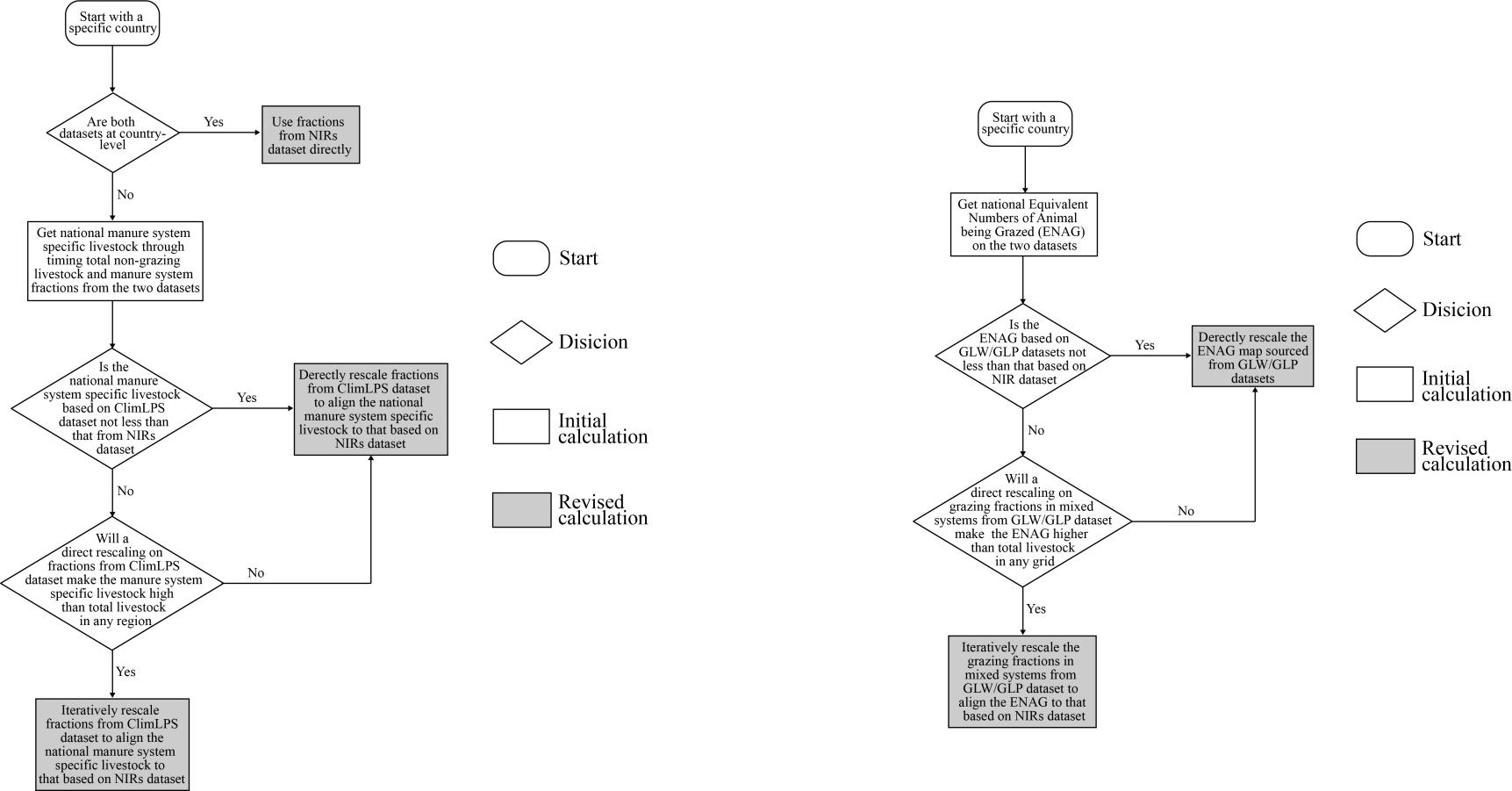


**Fig. S4** Decision making tree determining solid manure system fractions at the grid level


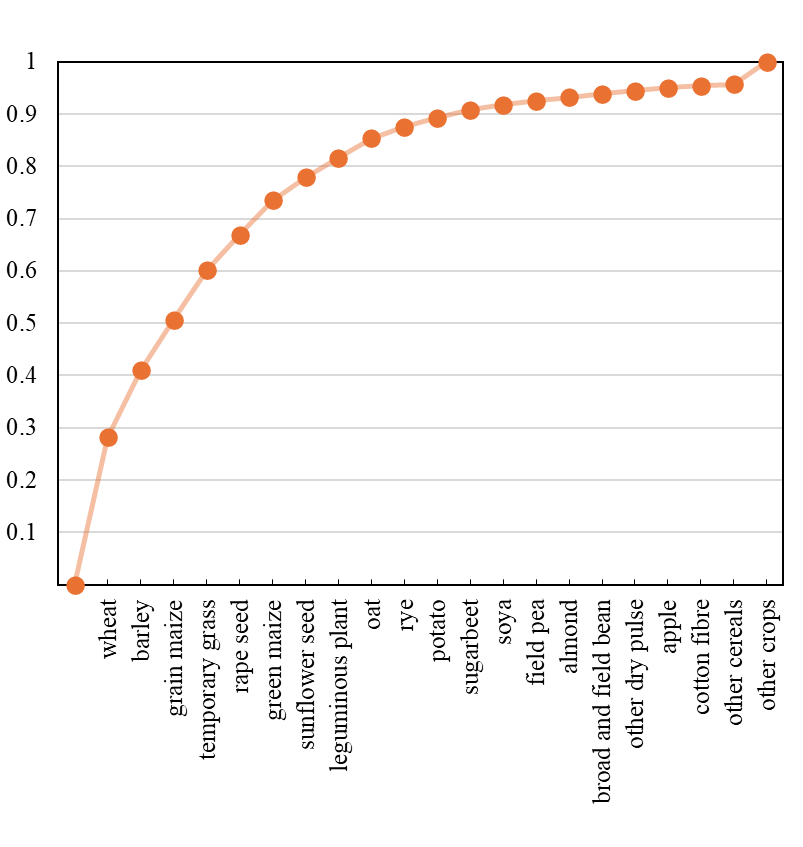


**Fig. S5** Cumulative proportion of specific crop areas (data source: Eustat^19^)

**Table S3.** Feed types and mapping schemes among datasets

| Feed types in our study | Feed type in Eurostat^19^ | Mapping scheme based on LUCAS^2^ or CROPGRID^1^ maps |
| --- | --- | --- |
| Temporary grassland | Temporary grasses (G1000) | Grassland (500) * Share_temporary_ |
| Permanent grassland | Permanent grassland (J0000) | Grassland (500) * Share_permanent_ |
| Legume fodder | Alfalfa (G2100)  Other legume (G2900) | Other fodder (excl. temporary grass) (250) * Share _legume_ |
| Green maize | Green maize (G3000) | Maize * Share _green maize_ |
| Other fodder | Other plants harvested green from arable land (G9000)  Other root crops (R9000) | Other fodder (excl. temporary grass) (250) * (1-Share _legume_) + other roots (223) |

Note: numbers in brackets are codes of land use types in corresponding datasets

**Table S4** Mapping table for crops in this study, fertilizer use by crops (FUBC)^5^ dataset and the MITTERA-EUROPE^15^ model

| Commodity names in this study | FUBC names | Crop groups for the manure allocation | Coefficients for the manure allocation |
| --- | --- | --- | --- |
| Wheat | Wheat | Group1 (G1) | 1 |
| Barley | Barley | Group1 (G1) | 1 |
| Maize | Grain maize, including corn cob maize | Group2 (G2) | 0.5 |
| Green maize | Silage maize | Group2 (G2) | 0.5 |
| Temporary grassland | Grassland* | Grass (Grass G1) | 1 |
| Legume fodder | Fodder (legumes) | Group4 (G4) | 0 |
| Rapeseed | Oilseed rape | Group1 (G1) | 1 |
| Sunflower | Sunflower, soya, linseed | Group2 (G2) | 0.5 |
| Soybean | Sunflower, soya, linseed | Group4 (G4) | 0 |
| Oats | Rye, triticale, oats, rice | Group2 (G2) | 0.5 |
| Rice | Rye, triticale, oats, rice | Group4 (G4) | 0 |
| Rye | Rye, triticale, oats, rice | Group2 (G2) | 0.5 |
| Potato | Potato | Group2 (G2) | 0.5 |
| Sugarbeet | Sugar beet | Group1 (G1) | 1 |
| Peas and beans | Pulses | Group4 (G4) | 0 |
| Nuts | Other crops | Group3 (G3) | 0.25 |
| Fruit | Permanent fruit | Group3 (G3) | 0.25 |
| Vegetable | Vegetables | Group2 (G2) | 0.5 |
| Permanent grassland | Grassland* | Grass (Grass G2) | 0.5 |

Note: * means ratios of permanent grass fertilizer use to temporary grass fertilizer use were taken from Einarsson et al.^6^

G1, G2 ,G3, G4, Grass G1 and Grass G2 are abbreviations of crop and grass groups for manure allocations, which correspond to abbreviations used in equations in our main text.

**Table S5** Country categories regarding grassland fertilization

| **Country groups** | **Characters of fertilization on grasses** | **Countries** |
| --- | --- | --- |
| Group 1 (GF1) | Only temporary grasses are fertilized | Bulgaria, Spain, Greece, Estonia, Finland, Hungary, Croatia, Netherlands, Lithuania, Latvia, Sweden, Romania, Portugal, the United Kingdom |
| Group 2 (GF2) | Ratios between temporary and permanent grasses could be obtained from Einarsson et al. directly | Belgium, Germany, Denmark, Ireland |
| Group 3 (GF3) | Ratios between temporary and permanent grasses could be calculated via information in Einarsson et al.^6^ and Ludemann et al.^5^ | Austria, Czech Republic, France, Italy, Poland, Slovakia, Slovenia, Norway, Switzerland |
| Group 4 (GF4) | No information, just refer to neighbouring countries | Cyprus,Malta,Luxembourg, Iceland, Liechtenstein |

Note: GF1, GF2 and GF3 are abbreviations of grassland fertilization groups, which correspond to abbreviations used in equations in our main text.

**Table S6** Regression functions estimating each items in ammonia models in Zhan et al.^17^

|  | **Rice** | **Upland crops** |
| --- | --- | --- |
| EF_SF0 | 0.132992 | 0.093408 |
| *f* (pH) | $\text{0.0224×}\text{e}^{\text{0.5555 ×pH}}$ | $\text{0.0429×}\text{e}^{\text{0.4955×pH}}$ |
| *f* (Temp) | $\text{0.0033×}\text{e}^{\text{0.2233}}\text{×Temp}$ | $\text{0.179×}\text{e}^{\text{0.094}}\text{×Temp}$ |
| *f* (Windsd) | $\text{0.2737×Ln Windsd+1.0605}$ | $\text{0.2737×Ln Windsd+0.9975}$ |
| *f* (Ftype) | $\sum_{\text{j}} \text{Prop}_{\text{j}}\text{×}\text{y}_{\text{j}}$  where $y_{j}=\left\{ \begin{aligned} \text{1.4797, j=ABC} \\ \text{1, j=Urea, }\text{AP,other}\text{ NP, NK,NPK} \\ \text{0.3345, j=AN,CAN,AS,AA,} \\ \text{ N solutions} \end{aligned} \right.$ | $\sum_{\text{j}} \text{Prop}_{\text{j}}\text{×}\text{y}_{\text{j}}$  Where$\text{ y}_{\text{j}}\text{=}$  $\left\{ \begin{aligned} \text{2.2910, j=ABC} \\ \text{1, j=Urea, AP,other NP, NK,NPK} \\ \text{0.5779, j=AN,CAN,AS,AA,} \\ \text{ N solutions} \end{aligned} \right.$ |
| *f* (Methd) | $\left\{ \begin{aligned} \text{1,}\text{ j=ABC,Urea,AP,other NP,} \\ \text{ NK,AN,}\text{C}\text{AN,AS} \\ \text{0}\text{.7326, j=AA, N solutions} \end{aligned} \right.$ | $\left\{ \begin{aligned} \text{1,}\text{ j=ABC, Urea,AP, other NP,} \\ \text{ NK, AN, }\text{C}\text{AN, AS} \\ \text{0}\text{.4966, j= N solutions} \\ \text{0}\text{.2477, j=AA} \end{aligned} \right.$ |

Note: EF_SF0 is the emission factors of synthetic fertilizers under the reference condition (chamber-based using urea applied through broadcasting with soil pH of 7 and air temperature of 20℃ for upland crops or of 26℃for rice). *f*(pH), *f*(Temp), *f*(Windsd), *f*(FType), and *f*(Methd) are emission modifying functions for soil pH, air temperature and wind speed during crop growth seasons (vary with crop types), and fertilizer application types and methods. Subscript *j* represents the type of synthetic fertilizer, including ABC(ammonium bicarbonate), AP(Ammonium phosphate), other NP (NP compound except AP), NK (NK compound), NPK(NPK compound), AN(ammonium nitrate), CAN(calcium ammonium nitrate), AS(ammonium sulfate), and AA(anhydrous ammonia). Prop_j_ is the proportion of synthetic fertilizer *j*.

**Table S7** Datasets used to update the ammonia models proposed by Zhan et al.^17^

| Variables | Datasets | Time | Ref |
| --- | --- | --- | --- |
| Soil pH | Harmonized World Soil Database (HWSD) v1.2 | No specific year | ^20^ |
| Temperature | Climatic Research Unit gridded Time Series (CRU TS) v4.07 | 2017 | ^21^ |
| Wind speed | TerraClimate | 2017 | ^22^ |
| Fertilizer type | IFASTAT | 2017 | ^23^ |
| Fertilization method | Eustat | 2010 | ^24^ |
| Crop growth period | USDA Foreign Agricultural Service;IFA Crop Calendars by Country | No specific year | ^25,26^ |


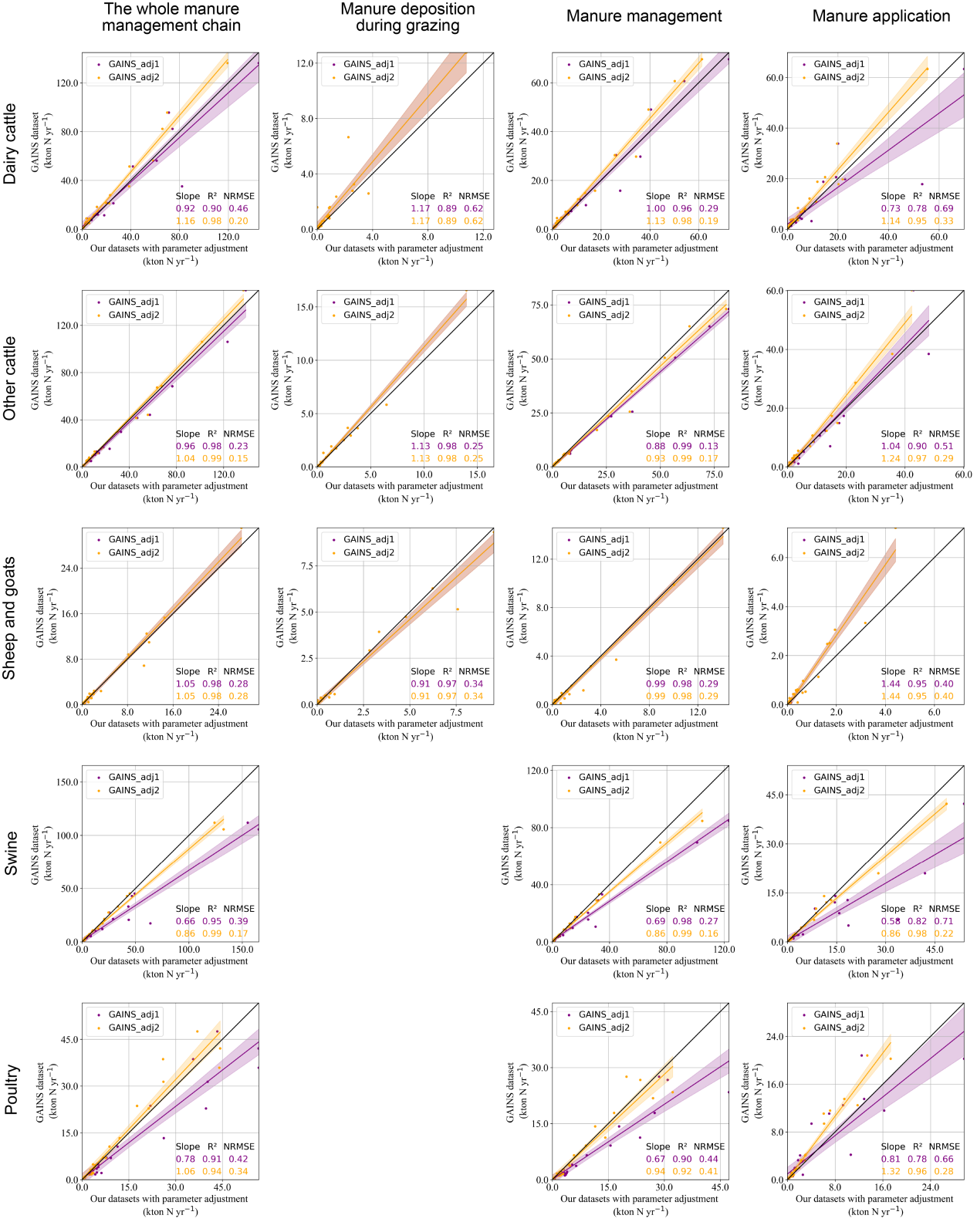


**Fig. S6** Comparisons of NH₃ emissions by livestock product and stage between our model—adjusted for GAINS excretion and emission factors (GAINS_adj1) and further modified with mitigation factors (GAINS_adj2)—and the original GAINS model.

**
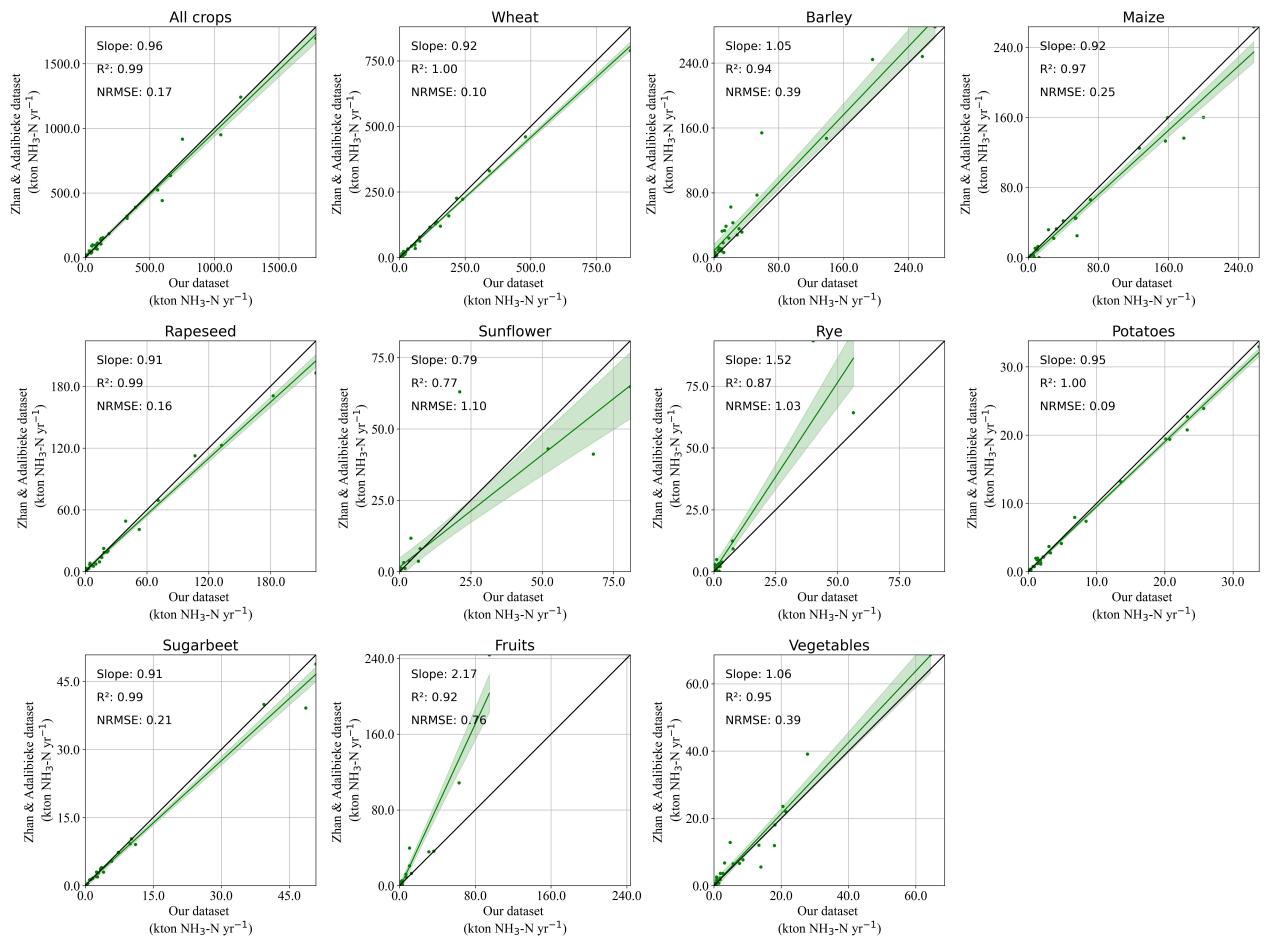
**

**Fig. S7** Comparisons of synthetic fertilizer N by crop product

**References**

1. Tang, F. H. M. *et al.* CROPGRIDS: a global geo-referenced dataset of 173 crops. *Sci Data* **11**, 413 (2024).

2. d’Andrimont, R. *et al.* From parcel to continental scale – A first European crop type map based on Sentinel-1 and LUCAS Copernicus in-situ observations. *Remote Sensing of Environment* **266**, 112708 (2021).

3. Copernicus Land Monitoring (CLMS) Grassland 2015 (raster 100 m), Europe, 3-yearly. https://doi.org/10.2909/b77b7ce3-04f8-44ae-aaae-b5e5af0f9682 (2018).

4. Gilbert, M. *et al.* Global livestock distribution in 2015 (5 minutes of arc). Harvard Dataverse https://dataverse.harvard.edu/citation?persistentid=doi:10.7910/dvn/sxhlf3 (2022).

5. Ludemann, C. I., Gruere, A., Heffer, P. & Dobermann, A. Global data on fertilizer use by crop and by country. *Sci Data* **9**, 501 (2022).

6. Einarsson, R. *et al.* Crop production and nitrogen use in European cropland and grassland 1961–2019. *Sci Data* **8**, 288 (2021).

7. *Global Livestock Production Systems*. (Food and Agriculture Organization of the United Nations, Rome, 2011).

8. UNFCCC. *National Inventory Submissions 2018 of Annex I Parties*. https://unfccc.int/process-and-meetings/transparency-and-reporting/reporting-and-review/reporting-and-review-under-the-convention/greenhouse-gas-inventories-annex-i-parties/submissions/national-inventory-submissions-2018 (2018).

9. Bioteau, T., Burton, C., Guiziou, F. & Martinez, J. *Qualitative Assessment of Manure Management in Main Livestock Production Systems and a Review of Gaseous Emissions Factors of Manure throughout EU27*.

10. Klimont, Z. & Brink, C. Modelling of Emissions of Air Pollutants and Greenhouse Gases from Agricultural Sources in Europe.

11. Velthof, G. L., Hou, Y. & Oenema, O. Nitrogen excretion factors of livestock in the European Union: a review. *Journal of the Science of Food and Agriculture* **95**, 3004–3014 (2015).

12. European Environment Agency. *EMEP/EEA Air Pollutant Emission Inventory Guidebook 2019: Technical Guidance to Prepare National Emission Inventories.* (Publications Office, LU, 2019).

13. Menzi, H. Manure management in Europe: results of a recent survey. in *Proceedings of the 10th International Conference of the RAMIRAN Network* (University of Veterinary Medicine, Slovak Republic, Štrbské Pleso, High Tatras, Slovak Republic, 2002).

14. Schulte-Uebbing, L. & de Vries, W. Reconciling food production and environmental boundaries for nitrogen in the European Union. *Science of The Total Environment* **786**, 147427 (2021).

15. Velthof, G. L. *et al.* Integrated assessment of nitrogen losses from agriculture in EU-27 using MITERRA-EUROPE. *Journal of Environmental Quality* **38**, 16 (2009).

16. Sommer, S. G., Webb, J. & Hutchings, N. D. New Emission Factors for Calculation of Ammonia Volatilization From European Livestock Manure Management Systems. *Frontiers in Sustainable Food Systems* **3**, (2019).

17. Zhan, X. *et al.* Improved Estimates of Ammonia Emissions from Global Croplands. *Environ. Sci. Technol.* **55**, 1329–1338 (2021).

18. Uwizeye, A. *et al.* Nitrogen emissions along global livestock supply chains. *Nat Food* **1**, 437–446 (2020).

19. European Union. Eurostat. European Union. Eurostat, https://ec.europa.eu/eurostat/data/database (2024).

20. FAO, International Institute for Applied Systems Analysis(IIASA), ISRIC-World Soil Information, Institute of Soil Science, Chinese Academy of Sciences (ISSCAS) & Joint Research Centre of the European Commission (JRC). Harmonized World Soil Database - Version 1.2.

21. Harris, I., Osborn, T. J., Jones, P. & Lister, D. Version 4 of the CRU TS monthly high-resolution gridded multivariate climate dataset. *Scientific Data* **7**, 109 (2020).

22. Abatzoglou, J. T., Dobrowski, S. Z., Parks, S. A. & Hegewisch, K. C. TerraClimate, a high-resolution global dataset of monthly climate and climatic water balance from 1958–2015. *Sci Data* **5**, 170191 (2018).

23. International Fertilizer Industry Association (IFA). IFASTAT database. (2024).

24. Eustat. Tilled arable area by tillage practice, EU-27, IS, NO, CH, ME and HR, 2010. https://ec.europa.eu/eurostat/statistics-explained/index.php?title=File:Tilled_arable_area_by_tillage_practice,_EU-27,_IS,_NO,_CH,_ME_and_HR,_2010.png&oldid=137925#file (2013).

25. US Department of Agriculture. USDA Foreign Agricultural Service. https://ipad.fas.usda.gov/rssiws/al/crop_calendar/europe.aspx (2024).

26. International Fertilizer Industry Association (IFA). FUBC Crop Calendars by Country. (2024).
