# Supplementary material for: Ammonia emissions from agricultural products at high resolution across Europe
Source: Sci Data. 2025 Aug 26;12:1493. doi: 10.1038/s41597-025-05110-9 (PMC12381134; doi:10.1038/s41597-025-05110-9)
Supplement: Supplementary file 2 — Supplementary Table 1 [file 41597_2025_5110_MOESM2_ESM.doc]

**Supplementary Table 1.** Variables and subscripts used for processing AP-AMMO

|  | **Definitions** |
| --- | --- |
| **Subscripts** |  |
| c | Crop type |
| k | Livestock type |
| g | Country |
| i | Grid cell |
| l | Land use type, including cropland(cl), grassland(gl), and agricultural land (al, equals cropland plus grassland) |
| t | Crop group differentiated by manure application rates |
| rsys | Livestock rearing system. Basic systems are grassland systems(gras), mixed system(mix), and other system(oth). The mixed systems are further classified to grazing (mix_graz) and non-grazing (mix_nongraz) parts. Grazing system (graz) equels ‘gras’ plus that in ‘mix_graz’. Non grazing system (non_graz) euqals ‘mix_nongraz’ plus ‘oth’. |
| msys | Manure management system, including solid (sld) and liquid (liq) systems |
| s | Stage of the manure management chain, including manure excretion (excr), manure deposition during grazing(depo), livestock housing (hous), manure storage(stor), and manure application(app). Since hous, stor, and app are consecutive three stages, we also set them as order 1, 2 , and 3 when tracing N and TAN flows in the manure management chain |
| **Variables** |  |
| AMI | Animal numbers |
| AMI_SD | Animal numbers derived from spatially explicit datasets |
| AMI_NIR | Animal numbers based on national level datasets |
| ENAG_SD | Equivalent numbers of animal being grazed based on spatially explicit datasets |
| ENAG_NIR | Equivalent numbers of animal being grazed based on national level datasets |
| Prop_SDmix_graz | Proportions of grazing animals in mixed systems |
| Prop_NIR_graz_ | Proportions of grazing animals based on national level dataset |
| Fac_scale | Scaling factors to make ENAG_SD equal ENAG_NIR |
| EXF | Manure excretion factors |
| N_Ma | Manure N flows |
| Prop_MS | Proportions of manure systems |
| Prop_TAN | Proportion of TAN to manure N |
| TAN_Ma | TAN in manure N flows |
| EF_Ma | Emission factor of NH_3_ from manure based on TAN |
| EF_Ma_oth | Emission factors of other gaseous N losses (incl. N_2_, N_2_O, and NO_x_) from manure based on total nitrogen |
| EMI_Ma | NH_3_ emissions sourced from manure |
| EMI_Ma_oth | Other gaseous N emissions (incl. N_2_, N_2_O, and NO_x_) from manure |
| AREA | Harvested areas of crops or grasses |
| Int_SF | Synthetic fertilizer N use intensities |
| Int_SF_v9 | Synthetic fertilizer N use intensities based on the newest FUBC dataset |
| Int_SF_vp | Synthetic fertilizer N use intensities based on previous FUBC datasets |
| N_SF | Total synthetic fertilizer N use for crops or grasses |
| Ratio_SF_PermTemp | The ratios of synthetic fertilizer use intensities for permanent grassland to those for temporary grassland |
| EF_SF | NH_3_ emission factors for synthetic fertilizer use |
| EMI_SF | NH_3_ emissions from synthetic fertilizer use |
| Prop_Ma_App | The proportions of manure applied to a specific land type |
| Int_Ma_App | The intensities of applied manure N |
| Coef_Ma_App | Coefficients of manure application rates for specific crop |
